# Tetramerization and interdomain flexibility of the replication initiation controller YabA enables simultaneous binding to multiple partners

**DOI:** 10.1093/nar/gkv1318

**Published:** 2015-11-28

**Authors:** Liza Felicori, Katie H. Jameson, Pierre Roblin, Mark J. Fogg, Transito Garcia-Garcia, Magali Ventroux, Mickaël V. Cherrier, Alexandre Bazin, Philippe Noirot, Anthony J. Wilkinson, Franck Molina, Laurent Terradot, Marie-Françoise Noirot-Gros

**Affiliations:** 1 Departamento de Bioquimica e Imunologia, Universidade Federal de Minas Gerais, UFMG, 31270-901, Belo Horizonte, MG, Brazil; 2 Sys2Diag FRE3690—CNRS/ALCEDIAG, Montpellier, France; 3 Structural Biology Laboratory, Department of Chemistry, University of York, York YO10 5DD, UK; 4 Synchrotron SOLEIL—L'Orme des Merisiers Saint-Aubin— BP 48 91192 GIF-sur-YVETTE CEDEX, France; 5 INRA, UMR1319 Micalis, F-78350 Jouy-en-Josas, France; 6 AgroParisTech, UMR1319 Micalis, F-78350 Jouy-en-Josas, France; 7 CNRS, UMR 5086 Bases Moléculaires et Structurales de Systèmes Infectieux, Institut de Biologie et Chimie des Protéines, 7 Passage du Vercors, F-69367 Lyon, France; 8 Université de Lyon, F-69622 Lyon, France; 9 Université Claude Bernard Lyon 1, F-69622 Villeurbanne, France

## Abstract

YabA negatively regulates initiation of DNA replication in low-GC Gram-positive bacteria. The protein exerts its control through interactions with the initiator protein DnaA and the sliding clamp DnaN. Here, we combined X-ray crystallography, X-ray scattering (SAXS), modeling and biophysical approaches, with *in vivo* experimental data to gain insight into YabA function. The crystal structure of the N-terminal domain (NTD) of YabA solved at 2.7 Å resolution reveals an extended α-helix that contributes to an intermolecular four-helix bundle. Homology modeling and biochemical analysis indicates that the C-terminal domain (CTD) of YabA is a small Zn-binding domain. Multi-angle light scattering and SAXS demonstrate that YabA is a tetramer in which the CTDs are independent and connected to the N-terminal four-helix bundle via flexible linkers. While YabA can simultaneously interact with both DnaA and DnaN, we found that an isolated CTD can bind to either DnaA or DnaN, individually. Site-directed mutagenesis and yeast-two hybrid assays identified DnaA and DnaN binding sites on the YabA CTD that partially overlap and point to a mutually exclusive mode of interaction. Our study defines YabA as a novel structural hub and explains how the protein tetramer uses independent CTDs to bind multiple partners to orchestrate replication initiation in the bacterial cell.

## INTRODUCTION

In all living organisms, chromosome replication is highly regulated to ensure only one initiation event per chromosome per cell cycle (1). Bacteria have developed various strategies to prevent inappropriate re-initiation, principally by regulating the activity and/or the availability of the master initiator protein DnaA. DnaA assembles at specific DNA sequences within *oriC* to promote the opening of the DNA duplex, and directs the assembly of the replisome machinery by first recruiting DNA helicase (1,2). DnaA is a member of the AAA+ (ATPases associated with diverse cellular activities) superfamily that binds to and hydrolyses adenosine triphosphate (ATP). Although both ATP and adenosine diphosphate (ADP) bound forms of DnaA are proficient in *oriC* binding, only the ATP-bound form is active in replication initiation (3).

In bacteria, multiple homeostatic mechanisms contribute to coordinate DNA replication with the cellular cycle (1,4). In Gram negative bacteria, proteins have been identified which regulate positively or negatively the initiation of replication by forming a complex with DnaA. Positive regulators such as DiaA in *E*scherichia *coli*, or the structural homolog HobA in *Helicobacter pylori* promote initiation by stimulating the assembly of ATP–DnaA at *oriC* (5–7). In *E. coli*, the major mechanism of initiation control is the regulatory inactivation of DnaA (RIDA), mediated by the ADP-activated protein Hda (homolog of DnaA) and the β-clamp subunit of the replicative DNA polymerase DnaN. The interaction between Hda, DnaN and ATP-bound DnaA, promotes the hydrolysis of ATP and the accumulation of inactive ADP–DnaA (8). RIDA has also been reported in *Caulobacter crescentus*, a Gram-negative proteobacteria phylogenetically distant from *E. coli* (9). In this bacterium, the Hda homolog HdaA prevents over-initiation and co-localizes with the replication machinery in the stalked cell. However, the presence of Hda seems to be restricted to proteobacteria suggesting that other bacterial species have developed different mechanisms for the negative regulation of DnaA.

In the Gram positive and spore forming bacterium *Bacillus subtilis*, replication initiation control is fulfilled by various proteins, which bind to DnaA and modulate its activity during the different life-styles of the bacilli. In cells committed to sporulation, the protein SirA prevents replication re-initiation by antagonizing DnaA binding to *oriC* (10–12). In vegetative cells, the primosomal protein DnaD and the ParA-like protein Soj were recently found to down regulate replication initiation by preventing the formation of a DnaA nucleofilament at *oriC* (13,14). The main regulatory protein YabA was also found to prevent over-initiation by inhibiting the cooperative binding of DnaA to *oriC* (13,14). Furthermore, YabA downregulates replication initiation as part of a multimeric complex with DnaA and DnaN that is associated with the replication factory (15–18). Thus, YabA is likely to act by trapping DnaA in a manner that can be both dependent and independent of DnaN during the cell replication cycle. However, although YabA, like Hda, regulates initiation through coupling to the elongation of replication, several pieces of evidence point to a distinct mode of action. In contrast to Hda, YabA has no sequence similarity to DnaA, and YabA does not belong to the AAA+ superfamily. YabA is a small protein of 119 residues, with an unusual predicted organization composed of a leucine zipper at its N-terminus and a putative zinc-binding domain at its C-terminus (15). Curiously, it lacks the classical bacterial DnaN-binding pentapeptide consensus motif (19). Given that YabA is conserved in low-GC Gram-positive bacteria, the protein represents the prototype of a distinct family of replication controller proteins.

To gain insight into the mechanism by which YabA interacts with multiple partners to control DNA replication, we have combined the X-ray structure of the N-terminal domain (NTD) of YabA, *in silico* modeling of the C-terminal domain (CTD) and small angle X-ray scattering (SAXS) of the full-length protein to derive a model of the YabA structure. The crystal structure of the NTD of YabA revealed an extended α-helix, four of which assemble into a helical bundle. We found that the CTD of YabA binds to a single Zn^2+^ ion with a zinc binding motif resembling known short Zn binding domains. Solution studies demonstrated that YabA is a tetramer in which the monomeric CTDs are separated from the tetrameric NTDs by a flexible linker resulting in an extended conformation. We then showed that the CTD is sufficient for interaction with either DnaA or DnaN. The interacting surfaces with DnaA and DnaN have been mapped within the CTD and unveil an atypical motif for binding to the β-clamp. Our study provides a structural explanation for the capacity of YabA to bind multiple partners, hereby defining a novel protein hub central to DNA replication in low GC Gram-positive bacteria.

## MATERIALS AND METHODS

### Proteins expression and purification

The *yabA* gene sequence was amplified from *B. subtilis* (strain 168) genomic DNA by polymerase chain reaction (PCR) and inserted into pET151/D-TOPO (Invitrogen) to generate the plasmid pET151-*yabA*. *E. coli* BL21 (DE3) cells (Invitrogen) carrying pET151-*yabA* were grown in LB medium (with ampicillin at 100 μg/l) at 37°C until OD_600_ = 0.6. Protein expression was induced with 1 mM isopropyl β-D-thiogalactopyranoside (IPTG) for 16 hours at 20°C. Cells were centrifuged and resuspended in buffer L (30 mM Tris pH 8, 150 mM NaCl, 1% Triton, 1 mM ZnCl_2_ and 5% Glycerol (V/V)) with protease inhibitor tablet (complete EDTA-free, Roche), lysozyme (Roche) and Dnase (Sigma-Aldrich). The cells were lysed by sonication and centrifuged at 16 000 *g* for 20 min. The soluble fraction was loaded on a HisTrap™ 5 ml column equilibrated with buffer A (30 mM Tris pH 8, 150 mM NaCl, 5% glycerol) and the protein eluted using a 0–100% gradient of buffer B (buffer A + 500 mM imidazole). Fractions containing YabA were incubated with TEV protease in the presence of 0.5 mM dithiothreitol (DTT) and 0.5 mM ethylenediaminetetraacetic acid (EDTA) and dialyzed for 16 h against buffer A at 4°C. The cleaved histidine tag was subsequently removed by passage of the dialysate through a HisTrap™ column. The flow-through fraction was concentrated and injected onto a Superdex 200 10/300 GL gel filtration column (GE Healthcare) equilibrated with buffer A. Fractions containing YabA were pooled and concentrated to 5 mg.ml^−1^.

To produce the YabA/DnaN complex, a vector (pET Lic_duet_yabA-dnaN) was used which directs the co-expression of N-terminally histidine tagged YabA and DnaN. *E. coli* BL21 (DE3) cells (Invitrogen) harboring (pET Lic_duet_yabA-dnaN) were grown in LB medium (with kanamycin at 40 μg/l) at 37°C until OD_600_ = 0.6. Protein expression was induced by addition of 1 mM IPTG and cells were incubated 3 h at 37°C. Cells were harvested by centrifugation and resuspended in buffer L with a protease inhibitor tablet (complete EDTA-free, Roche), lysozyme (Roche) and Dnase (Sigma-Aldrich). The cells were lysed by sonication and centrifuged at 16 000 *g* for 20 min. The soluble fraction was diluted four times with buffer A and applied on a HisTrap™ 5 ml column equilibrated with buffer A. The protein complex was eluted using a 0 to 100% gradient of buffer B. Fractions containing the YabA/DnaN complex were pooled and applied to a Superdex 200 10/300 GL gel filtration column (GE Healthcare) equilibrated with buffer A. The pure YabA/DnaN complex was concentrated to 5 mg ml^−1^.

For the preparation of YabA^1–58^ and YabA^70–119^, the coding region of *yabA* was amplified from *B. subtilis* genomic DNA (strain 168) by PCR using gene-specific primers, with appended sequences to facilitate ligation independent cloning. The PCR product was inserted into the vector pET-YSBLIC3C by ligation-independent cloning methods (20). Plasmids encoding N- and C-terminal fragments of YabA, residues 1–58 (YabA^1–58^) and residues 70–119 (YabA^70–119^) respectively, were created using a deletion mutagenesis method. A whole vector amplification of pET-YSBLIC3C-YabA was performed by PCR using the primers listed in Supplementary Table S5. The recombinant plasmids pET-YSBLIC3C-YabA^1–58^ and pET-YSBLIC3C-YabA^70–119^ direct the expression of YabA^1–58^ or YabA^70–119^, respectively, each fused to a 3C cleavable N-terminal His-tag. The recombinant YabA fragments were overproduced in *E. coli* BL21 (DE3) and following cell lysis, purified by steps of immobilized nickel affinity and gel filtration chromatography similar to those described above with the affinity tag subsequently being removed by HRV 3C protease.

### Crystallization, structure determination and refinement

Two crystals forms of YabA were obtained using vapor diffusion methods in hanging drops consisting of 1.0 μl of protein or protein complex and 1.0 μl of reservoir solution containing 20% PEG 3350 (W/V), 100 mM Bis-Tris propane pH 6.5 and 200 mM potassium thiocyanate. Crystal form I was obtained using the purified YabA/DnaN complex (5 mg.ml^−1^). A second crystal form (II) was obtained using YabA (5 mg.ml^−1^) and by including 0.025% (V/V) dichloromethane in the drop. For structure determination, form II crystals were soaked in reservoir solution supplemented with 10 mM platinum potassium thiocyanate for 16 h. Crystals were then flash-cooled in liquid nitrogen (100K) using mother liquor containing step-wise additions of glycerol (15% final concentration) as cryoprotectant. X-ray diffraction data were collected at the ID14EH4 (form I) and ID23EH1 (form II) beamlines of the European Synchrotron Radiation Facility (ESRF, Grenoble). Form I crystals belonged to the space group P3_2_21 with unit cell dimensions of a = b = 83.4 Å and c = 64.7 Å and diffracted to a resolution of 2.7 Å. Crystal form II belonged to the space group P6_1_22 with unit cell dimensions of a = b = 83 Å and c = 110 Å and diffracted to a resolution of 4 Å according to CC_1/2_ (21). The diffraction data were indexed and integrated using XDS (22) and scaled with SCALA from the CCP4 program suite (23). Data collection statistics are given in Table 1.

**Table 1.** Data collection, phasing and refinement statistics

	Native	Pt derivative
ESRF beamline	ID14EH4	ID23EH1
Wavelength (Å)	0.93222	1.0717 Å
Space group	P 3_2_ 2 1	P 6_1_ 2 2
Unit cell parameters (Å)		
	a = 83.48	a = 83
	b = 83.48	b = 83
	c = 64.71	c = 110
Number of protein molecules per asymmetric unit	2	2
Resolution range (Å)	36.1–2.7	43.69–4.0
	(2.8–2.7)	(4.2–4.0)
Completeness (%)	98.5 (99.9)	99.9 (100.0)
I/σ(I)	18.02 (1.24)	11.5 (2.6)
No. of measured reflections	51889 (5561)	45757 (6834)
Redundancy	7.1 (7.4)	21.2 (23.0)
Anomalous multiplicity		12.7 (13.0)
R_SYM_ (%)	7.3 (262.6)	14.1 (145.0)
CC_1/2_ (%)	99.9 (52.7)	100 (74.6)
Heavy atom sites		2
FOM (after Phaser)		0.24
FOM (after Resolve)		0.58
R_work_/R_free_ factor (%)	22.23/27.01	
N^o^. of non hydrogen atoms		
Macromolecules	1063	
Water	4	
Wilson B-factor (Å^2^)	90.1	
Average B-factor (Å^2^)	126.6	
RMSD		
Bonds (Å)	0.005	
Angles (°)	0.616	
Ramachandran (%)		
Favored region	97.5	
Allowed region	2.5	
Outliers region	0.0	

A single high redundancy dataset was collected at the Pt edge on a form II crystal soaked into platinum thiocyanate. The structure was solved using Autosol from the PHENIX package (24). The experimental map obtained at 4 Å and the two Pt sites identified were used in Autobuild which resulted in a non crystallographic symmetry (NCS) averaged, solvent flattened and improved map (24). The initial model consisted of two polyalanine helices of 30 residues and was subsequently extended manually in the improved low-resolution experimental map. The model was then used for molecular replacement using the data obtained from crystal form I. The model was built manually using COOT (25) and refined using PHENIX (24). Phasing and refinement statistics are indicated in Table 1. Figures depicting the structure were generated with PyMOL (26). The coordinates of YabA^1–62^ have been deposited in the protein databank (pdb code 5DOL).

### SEC-MALS

Samples (100 μl) of YabA, YabA^1–58^ and YabA^70–119^ were loaded onto gel-filtration columns equilibrated with 50 mM Tris pH 8.0, 150 mM NaCl. YabA was loaded at a concentration of 2.5 mg.ml^−1^ onto a Superdex 200 HR 10/30 column and YabA^1–58^ and YabA^70–119^ were loaded at concentrations of 5 mg.ml^−1^ onto a Superdex 200/75 HR 10/30 column. In each case, the column eluate was successively analyzed by a SPD20A UV/Vis detector, a Wyatt Dawn HELEOS-II 18-angle light scattering detector and a Wyatt Optilab rEX refractive index monitor. Data were analyzed with Astra software (Wyatt).

### Circular dichroism

Circular dichroism spectra were recorded at 20°C on a Jasco J-810 CD spectrophotometer using a quartz cell with a 0.1 cm path length. Experiments were carried out at protein concentrations of 0.2 mg.ml^−1^ in a buffer of 20 mM potassium phosphate, pH 8.0. Spectra were recorded across the wavelength range of 260–185 nm. A buffer scan was also recorded and subtracted from the protein spectra to remove any contribution from the buffer. Analysis of YabA CD spectra was performed using Dicroweb (http://dichroweb.cryst.bbk.ac.uk) (27).

### Atomic absorption spectroscopy

For AAS we used a Phillips PU9200 double beam spectrometer equipped with a flame volatilization system. Samples of YabA dissolved in deionized water at 0.5 mg.ml^−1^ were analyzed in triplicate using a zinc-specific lamp of wavelength 213.9 nm and the absorbance values were compared to those for a standard curve derived from samples of zinc powder dissolved in HCl.

### *In silico* molecular modeling of CTD

Template Search and Secondary Structure Prediction: the amino acid sequence analysis was performed using the Position Specific Iterated BLAST (PSI-BLAST) and Pattern Hit Initiated BLAST (PHI-BLAST) methods at NCBI (http://www.ncbi.nlm.nih.gov/BLAST/) and using the All Non-Redundant (NR) amino acid sequence database from April 2000, which includes SwissProt, CDS translation of GenBank (gb), EMBL (emb), the DNA database of Japan (dbj) and the Protein Structure Database (pdb) (28). Default amino acid replacement matrices and gap penalties were used in all database searches. Secondary structure predictions were made using JPRED (http://www.compbio.dundee.ac.uk/jpred) through the Jalview version 2 software (29).

Full length YabA and two fragments encompassing residues 1–70 and 71–119 were scanned against the protein databank (PDB) by PSI-BLAST over five iterations. PHI-BLAST was also used to search templates for the C-terminal part of YabA. The templates selected by the BLAST approach were aligned with YabA sequence using ISPALIGN (Intermediate Sequence Profile Alignment) (29). A manual alignment adjustment was performed with the help of Geneious Pro 4.8.4 software (Biomatters Ltd). The identity and similarity between YabA-CTD and its templates (aminoacids 76–119) were computed using SIAS tool (http://imed.med.ucm.es/Tools/sias.html) with a BLOSUM62 matrix. Based on alignment results, a 3D model of YabA was obtained by homology modeling using the software package Modeller 9v8 (30). Image rendering and H95 position refinement were performed using PyMOL (DeLano Scientific LLC) sculpting properties. The Qmean package was used to evaluate model quality (http://swissmodel.expasy.org/qmean) (31,32).

### SAXS data collection

Protein samples were centrifuged for 10 min at 10 000 rpm prior to X-ray analysis in order to eliminate all aggregates. Sample concentration was measured by UV absorption at λ = 280 nm on a Thermo Scientific NanoDrop 1000 Spectrophotometer. For each sample, a stock solution was prepared at a final concentration of 8 mg/ml and stored at 4°C and then directly used for the experiments. SAXS experiments were conducted on the SWING beamline at the SOLEIL synchrotron (λ = 1.033 Å). The Aviex charge-coupled device detector was positioned to collect data in the q-range 0.006–0.5 Å^−1^ (Q = 4πsinθ.λ^−1^, where 2θ is the scattering angle). The solution was injected in a fixed-temperature (15°C) quartz capillary with a diameter of 1.5 mm and a wall thickness of 10 μm, positioned within a vacuum chamber. A total of 80 μl of monodisperse protein sample were loaded onto a size-exclusion column (SEC-3 300 Ǻ Agilent), using an Agilent HPLC system and eluted directly into the SAXS flow-through capillary cell at a flow rate of 0.2 ml min^−1^ (33). The elution buffer consisted of 50 mM Tris–HCl pH 8, 150 mM NaCl, 5% glycerol filtered and degassed. SAXS data were collected continuously, with a frame duration of 1.5 s and a dead time between frames of 0.5 s. Data reduction to absolute units, frame averaging and subtraction were done using FOXTROT, a dedicated home-made application. All subsequent data processing, analysis and modeling steps were carried out with PRIMUS and other programs of the ATSAS suite (34).

### SAXS data processing, analysis and molecular modeling

The experimental SAXS data for all samples were linear in a Guinier plot of the low *q* region, indicating that the proteins had not undergone aggregation. The radius of gyration *R_g_* was derived by the Guinier approximation *I*(*q*) = *I(*0) exp(-*q*^2^*R*_g_^2^/3) for *q*R_g_ < 1.0 using PRIMUS (35). The program GNOM (36) was used to compute the pair-distance distribution functions, *p(r)*. This approach also features the maximum dimension of the macromolecule, *D*_max_. The overall shapes of the entire assemblies were derived from the experimental data using the program GASBOR (37). These models were averaged to determine common structural features and to select the most typical shapes using the programs DAMAVER (38) and SUPCOMB(39). An initial model of the YabA tetramer was built by assembling a tetramer of the NTD (residues 1–61) as seen in the crystal structure with four homology models of the CTD (residues 76–119). A linker of 14 residues between each NTD and CTD generated a complete full-length tetramer. To generate a model of full-length YabA using the crystal structure, and the homology model, which was compatible with the SAXS data, we performed atomic modeling using Dadimodo (40), a genetic algorithm based rigid-body refinement analysis program. The NTD and CTD structures were not modeled further but the linker region of YabA and the positions of the four CTDs were allowed to move. Continuity of the structure was assured by subsequent energy minimization using Dadimodo (40). A SAXS χ^2^ value was then computed for each eligible structure, using CRYSOL. The averaged scattering curve of YabA and the fitting curve calculated from the model produced by Dadimodo were superimposed with CRYSOL (41).

### Yeast two-hybrid and three-hybrid assays

Full length YabA and the C-terminal region (62–119), full length DnaA and an N-terminally truncated DnaA fragment (71–440), and DnaN proteins were expressed as fusions to the GAL4 binding domain BD or activating domain AD from the vectors pGBDU-C1 and PGAD-C1, respectively. pGBDU and PGAD derivative constructs were introduced by transformation into PJ694- (α) and (a) haploid strains, respectively. Binary interactions were tested by combinational mating of the strains expressing the BD and AD fusions as previously described (15). Interacting phenotypes are tested by the ability of the diploid cells to grow on selective media SD-LUH and SD-LUA. For 3HB experiments, DNA fragments encoding full length YabA or the C-terminal domain were inserted into the p3HB vector (18). The p3HB-YabA derivatives were co-expressed with either DnaA or DnaN in haploid (α) strains and the strains expressing the different combination pairs are then mated again with haploid (a) strains expressing the appropriate AD-fusion.The ability of a given BD-protein fusion to interact with a given AD-protein fusion conditional upon the presence of a third protein is attested by the appearance of growth on SD-LUWH and SD-LUWA selective media as previously described (18).

### Site targeted mutagenesis and analysis of Loss-of-Interaction (LOI) phenotypes by yeast-two hybrid

Site-directed saturation mutagenesis of *yabA* was performed by PCR amplification and fragment joining using degenerate oligonucleotides containing a randomized codon at the targeted position. The mutated PCR amplified *yabA* coding sequences were cloned into the pGBDU vector, in frame with the BD domain of GAL4 as previously described (15). Mutations were identified by sequencing and pGBDU-*yabA* derivatives carrying mutations at the targeted codon leading to different amino-acid substitutions were introduced into the pJ69–4 (a) yeast haploid strain by transformation. Yeast haploid cells expressing the BD-*yabA* mutant derivatives were mated with haploid strains of the opposite mating type (pJ69–4 (α)), harboring the pGAD-partner constructs encoding interacting proteins DnaA, DnaN or YabA fused to the AD domain of GAL4, as previously described (15). Interacting phenotypes were assessed according to the ability of the diploid forms to grow on (-LUH and -LUA) selective media. Mutants were tested for their ability to express interacting phenotypes with each of the three partners. The screening for loss-of-interaction (LOI) mutations against a single partner while maintaining the ability to bind to the other partners precludes the isolation of mutations that destabilize the tertiary structure of YabA.

### Construction of GFP fusions

Wild-type and mutant *yabA* genes were PCR amplified from the corresponding pGBDU-*yabA* constructs for subsequent cloning into the vector pGS1190 to generate *cfp-yabA* fusions under the control of the xylose-inducible promoter P_xyl_ (42). The *cfp-yabA* constructs were then integrated into the *amyE* locus of the *B. subtilis* Δ*yabA* strain, JJS142 (Supplementary Table S4). For the fluorescence complementation assay, genomic DNA from a strain expressing the *yfp-yabA-N85D* fusion under the control of its native promoter at the locus position, (17) was used to transform the recipient strains carrying the xylose inducible *cfp-yabA* constructs at *amyE* locus (see Supplementary Table S4), using chloramphenicol (5μg/ml) for selection.

### Fluorescence microscopy

Cells expressing the CFP-tagged YabA mutant derivatives and cells co-expressing CFP-YabA and YFP-YabA were grown and treated as previously described (15). For microscopic observations, the cells were mounted on agarose slides (43). Images were acquired by using an HQ snap digital charge-coupled device camera and analyzed using Metamorph v3 software. Appropriate filter sets to visualize the CFP and YFP fluorescence signals were obtained from Leica/WWR. DNA was stained with DAPI, and membranes were stained with FM5–95 (Molecular Probes).

## RESULTS

### YabA is a tetrameric, multidomain Zn-binding protein

**Figure 1.**
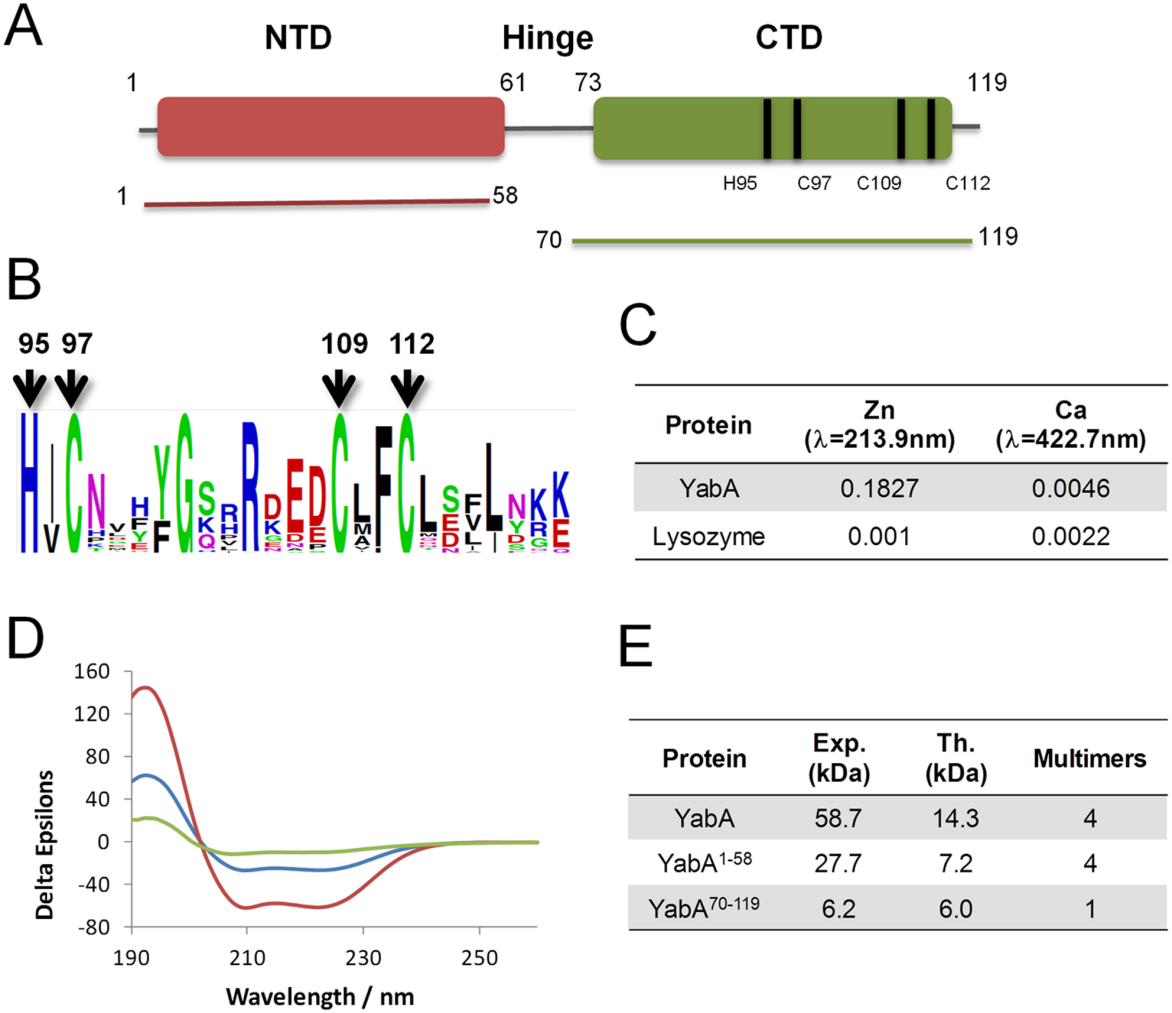
(**A**) Schematic representation of YabA domain organization with the NTD (Indian red) and CTD (green) protein constructs used in the study. Bars indicate the position of Zn binding residues. (**B**) Web logo showing conservation of amino acid residues in the C-terminal region of YabA. Conserved histidines and cysteines are indicated by arrows. (**C**) Atomic Absorption Spectroscopy of YabA in solution. Zn measurements by AAAF. (**D**) Circular dichroism spectroscopy. Spectra were recorded for YabA (blue), YabA^1–58^ (red) and YabA^70–119^ (green). All three spectra exhibit features characteristic of a folded protein, with increasing molar ellipticity with decreasing wavelength below 205 nm and minima over the wavelength range 205–225 nm indicating a preponderance of α-helices. (**E**) Molecular mass measured from SEC-MALS analysis.

Homology-based sequence analysis suggested that YabA consists of N-terminal (residues 1–56) and C-terminal (residues 73–119) structural domains separated by a region of low complexity and poorly conserved sequence, which we will refer to as a ‘linker’ or ‘hinge’ (Figure 1A, S1). The N-terminal region includes a conserved leucine heptad repeat (residues 6–48) and a regular distribution of charged residues, pointing to a potential leucine zipper-like domain. The C-terminal region exhibits higher sequence conservation, with three cysteine residues (C97, C109, C112) and one histidine (H95) being invariant in YabA sequences, suggesting they participate in the coordination of a zinc ion (Figure 1A and B and Supplementary Figure S1). A PSI-BLAST search of non-redundant sequence databases using the C-terminal region of YabA as seed allowed us to assign a YabA-like function to 46 additional proteins harboring the HCCC motif in firmicutes (Supplementary Table S1).

The presence of zinc atoms in purified *B. subtilis* YabA was thus studied by spectroscopic methods. Atomic absorption spectra of the purified protein dissolved in deionized water revealed strong absorption at 231.9 nm diagnostic of the presence of zinc (Figure 1C). Quantitative measurements calibrated against a zinc standard curve gave a zinc:YabA molar ratio of 0.94 indicating that one zinc ion is bound per YabA chain. These data suggest that YabA defines a novel class of bacterial Zn binding proteins in which the HCCC motif could be used to coordinate one Zn per protein chain.

To assess the overall secondary structure composition, circular dichroism spectra were recorded for purified YabA, as well as NTD and CTD recombinant fragments, YabA^1–58^ and YabA^70–119^ (Figure 1D). The spectra for all three species exhibit shallow minima in their molar ellipticity over the wavelength range 205–225 nm and increasing molar ellipticity with decreasing wavelength below 205 nm. These strong secondary structure characteristics indicate that the proteins are folded. Analysis of these spectra using Dicroweb confirmed this conclusion and indicated that the secondary structures of full length YabA and its domain fragments feature a high percentage of α-helices. To determine the quaternary structures of YabA, YabA^1–58^ and YabA^70–119^, the proteins were next analyzed by size exclusion chromatography and multi-angle laser light scattering (SEC-MALS). In these experiments, the protein samples are analyzed on a gel-filtration column and the absorbance at 280 nm and the refractive index of the eluting species are monitored together with the multi-angle laser light scattering of the sample. YabA eluted as a single peak from an S200 column with a retention time of ∼26.5 min (Figure 1E and Supplementary Figure S2) and a molecular mass of 58.7 kDa. As the theoretical molecular mass of YabA is 14.3 kDa, we concluded that recombinant YabA is a tetramer (57.2 kDa) in solution as previously reported (15). The N-terminal fragment YabA^1–58^ had a retention time of ∼21.5 min on an S75 column and this peak was associated with an Mw of 27.7 kDa (Figure 1E and Supplementary Figure S2). This value is in agreement with the mass calculated for a YabA^1–58^ tetramer (28.8 kDa). Interestingly, YabA^70–119^ eluted from the same column as a single peak with a retention time of ∼28 min and an associated Mw of 6.2 kDa (Figure 1E, Supplementary Figure S2), indicating a monomeric state for this C-terminal domain fragment (theoretical molecular mass of 6.0 kDa). We conclude that YabA tetramer formation relies predominantly on the N-terminal portion of the molecule.

### Crystal structure of YabA NTD

**Figure 2.**
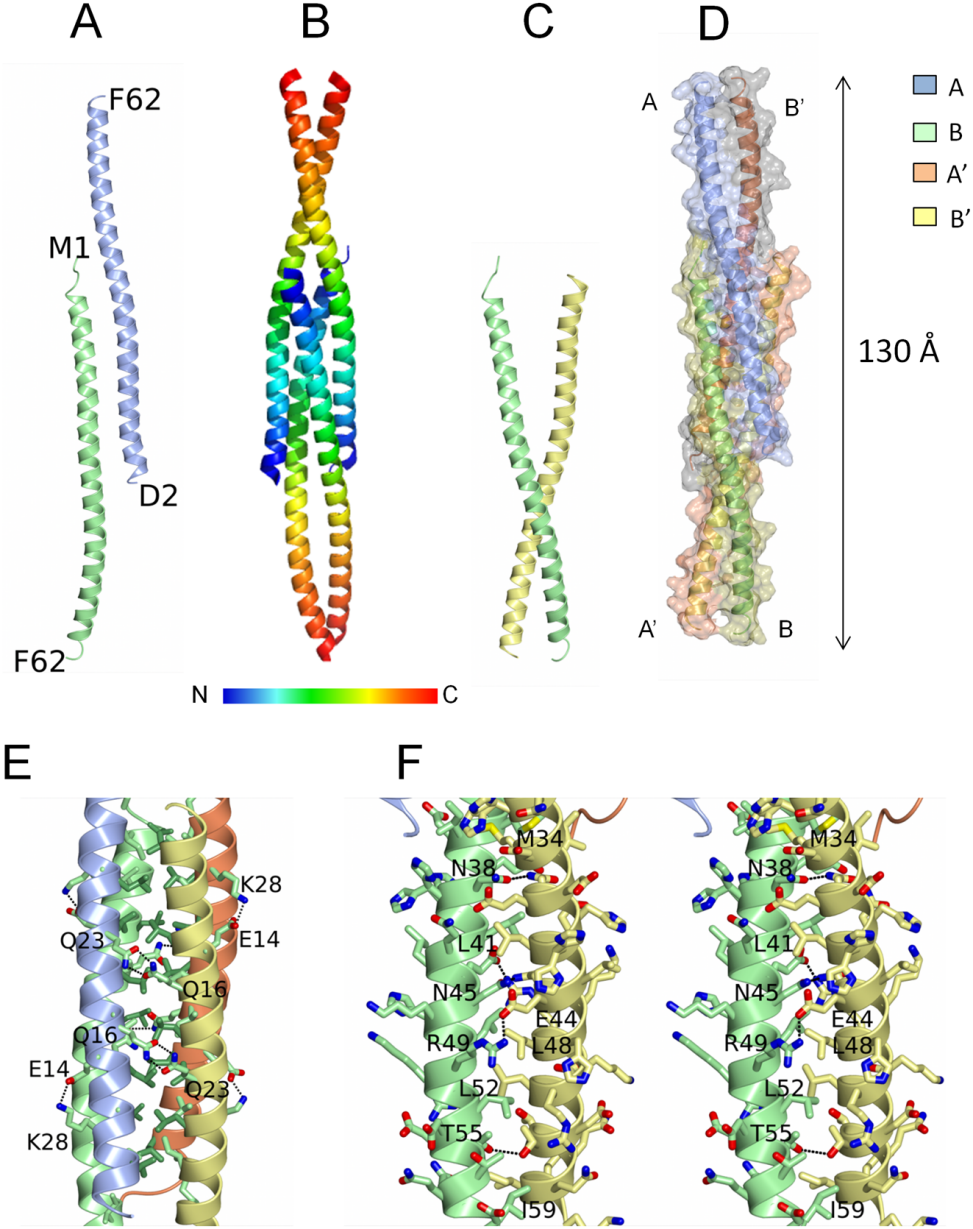
Structure of the YabA-NTD tetramer. (**A**) Ribbon representation of the two chains of YabA^1–62^ in the asymmetric unit A (ice blue) and B (light green) with the N- and C-terminal residues labelled. (**B** and **D**) The YabA^1–62^ tetramer. Each chain is shown as a ribbon either colored ramped as shown in the key (B) or colored by chain (D) with an accompanying surface rendering. (**C**) Ribbon representation of molecules A’ and B illustrating the tweezer arrangement of these chains. (**E**) The four helix bundle region. The chains are displayed as ribbons colored by chain A (ice blue) B (light green) A’ (lemon) and B’ (coral). The Cα and side chains of apolar residues in the interior are colored by residue type, light green for Val and Ile and lawn green for Leu to emphasize the leucine zipper. Polar interactions between the chains are shown as dashed lines and residues forming the prominent interchain interactions referred to in the text are labeled. (**F**) Stereo image of the coiled coil region of chains B and A’. The Cα and the side chains of residues from the two chains are displayed with the carbon atoms colored according to the chain and with nitrogens in blue, oxygens in red and sulphurs in yellow. Polar interactions between the chains are shown as dashed lines.

We attempted to crystallize YabA and obtained two crystal forms (form I and II, see ‘Materials and Methods’ section for details). In both cases, analysis of the unit cell contents and lattice interactions indicated that proteolysis occurred during crystallization. The final model allowed us to delineate the NTD boundary of the crystallized fragment as residing around residue F62 in the form I crystals, fragment that will be thus described as YabA^1–62^. The Form I and II crystals each contain two molecules of YabA^1–62^ per asymmetric unit arranged almost identically. The structure obtained with form I crystals was refined to a final resolution of 2.7 Å with good geometry (Table 1). The YabA^1–62^ protomer consists of a single 90 Å long α-helix comprising 15 turns with a pronounced curvature at its center (Figure 2A). YabA^1–62^ assembles as a dimer of dimers in the crystal consisting of chains A and B and their symmetry equivalents A’ and B’ (Figure 2B). The A'B dimer resembles a pair of tweezers (Figure 2C) assembled via tight interactions of the C-terminal elements of the two helices whose arrangement can be described as a parallel coiled-coil. Two pairs of these tweezers (AB’ and A'B) embrace each other to form the head-to-head tetramer (Figure 2D). The tetramer thus comprises a central four-helix bundle flanked by pairs of helical extensions arranged as coiled coils (Figure 2B and D). The packing of the molecules appears to be tight with ∼40% of the surface area of each chain buried by intermolecular interactions, with each subunit forming extensive contacts with its three neighbors. As a result, there is a total buried surface area of 9880 Å^2^ in the tetramer. The four-helix bundle comprises residues 1–35 of the four chains with chains A and B’ running antiparallel to chains A’ and B. The interior of the bundle is formed by the close packing of the aliphatic side chains of L6, V10, L13, I17, L20, L24, L27, I31 and M34 from all four chains creating a substantial hydrophobic core (Figure 2E). The heptadic arrangement of L6, L13, L20 and L27 gives rise to a leucine zipper-type interdigitation of side chains projecting from pairs of adjacent chains (A-A’ and B-B’). Sets of reciprocal charge-charge interactions (four in total) between the E14 and K28 side chains on the A chains and hydrogen bonds between Q16 and Q23 side chains on the B chains presumably lend further stability to the tetramer (Figure 2E).

The flanking coiled-coil regions are formed by the C-terminal halves of chains A and B’ on one side of the four helical region and chains A’ and B on the other (residues 36–62). These pairs of helices, which are splayed in the four helix bundle region, converge at their C-termini (Figure 2C) where hydrophobic interactions of the side chains of M34, L41, L48, L52 and I59 with the equivalent residues in the partner chain are augmented by hydrogen bonding interactions of the side chains of N38, N45, T55 and most strikingly by reciprocal salt-bridging interactions between the side chains of E44 and R49 (Figure 2F). The crystal structure of YabA^1–62^ is consistent with the circular dichroism data which suggested a high proportion of α-helix in the YabA^1–58^ fragment and the SEC-MALS data which clearly point to a tetramer. The structure also shows that residues 59–61 previously assigned to the linker region can adopt a α-helical conformation.

### YabA CTD *in silico* structure model

**Figure 3.**
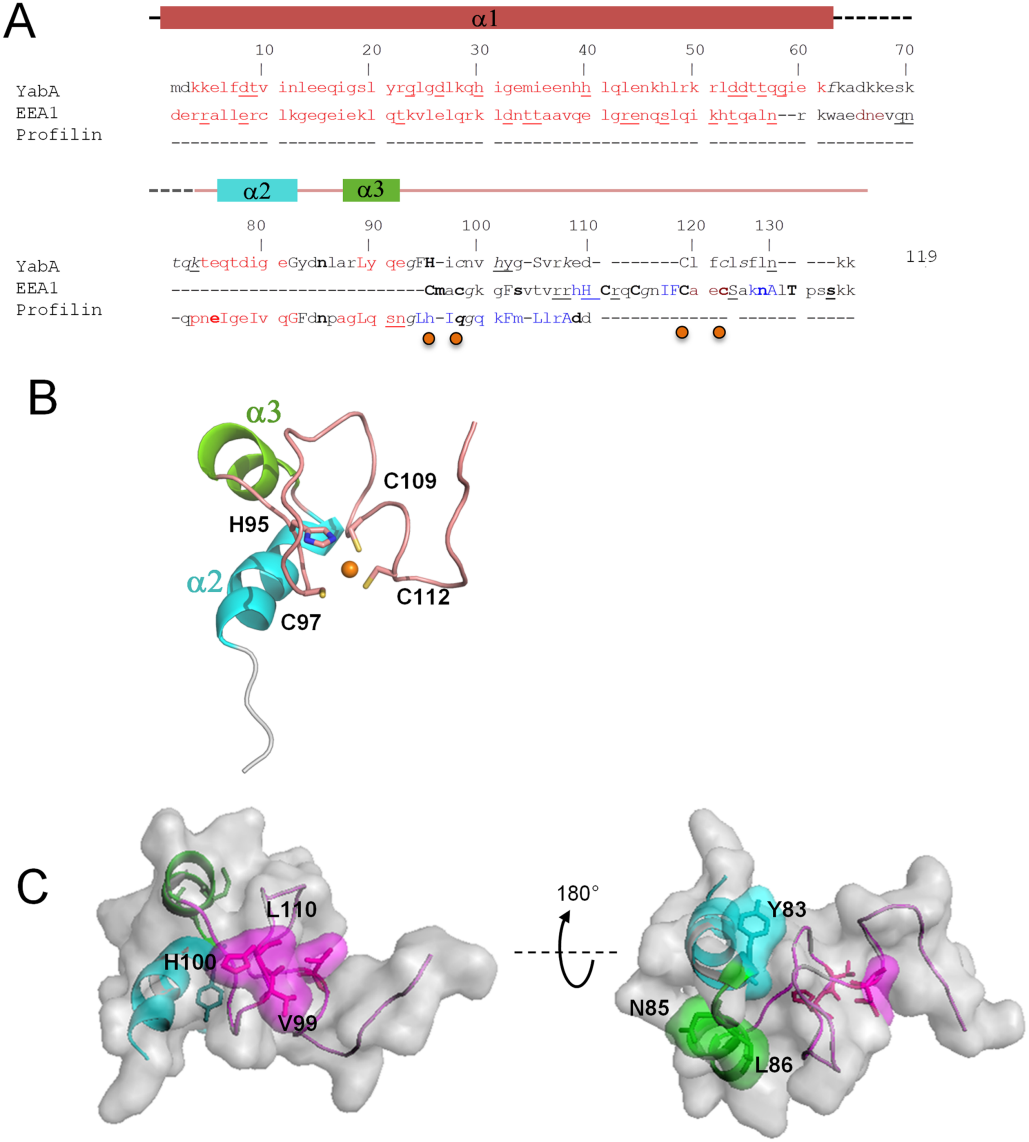
Homology model of the YabA-CTD monomer. (**A**) Structural sequence alignment of YabA model with the identified homologs EEA1 (pdb code 1JOC) (46) and profilin (1YPR) using the Joy algorithm(63), highlighting the structural similarities between YabA model and it's templates. Alpha helix (red); 3^10^ helix (maroon); beta strand (blue); solvent accessible residues (lower case); solvent inaccessible residues (upper case); hydrogen to bond main-chain amide(bold), hydrogen bond to main-chain carbonyl (underline). Secondary structures of YabA are indicated on top and correspond to the crystal structure (residues 1–62) and the homology model (76–119). The residues involved in Zn coordination are indicated by orange dots. (**B**) Cartoon representation of YabA CTD model colored as in (A). Side chains of the residues participating in Zn binding are indicated as ball and sticks. (**C**) Surface representation of YabA-CTD revealing surface-exposed residues important for interaction with DnaN (left) and DnaA (right) identified in a previous study (15).

In the absence of a crystal structure for the CTD, we searched for relevant structural templates to build a three-dimensional model of YabA CTD. Using a PSI-BLAST search we first identified *Saccharomyces cerevisiae* profilin (pdb code: 1YPR) which exhibited partial similarity to YabA CTD although it does not contain a zinc finger motif (44). In order to refine the template identification for the zinc finger motif, a hypothesis-driven pattern search was performed. Protein of known structures containing variations of the zinc-binding motif of YabA H-x-C-x(11)-C-x(2)-C were identified using PHI-BLAST (45). These include a series of zinc finger proteins that belong to the PHD, RING, CHY and FYVE domain families (Supplementary Table S2). The structure of the endosome targeting protein EEA1 (pdb code: 1JOC) (46) was of particular interest since the sequence similarities extend beyond the FYVE zinc-binding domain to the linker region and the NTD of YabA (Figure 3A and B). Based on a multiple alignment of the combination of homologous regions of interest from the structural templates, a 3D-model of YabA CTD was built using constraint-based homology modeling procedures implemented in Modeller 9v8 (24) (Figure 3B). To evaluate the quality of its fit to experimental structures the model was next validated by QMEAN (32). The determined Qmean Z-scores were -1.59 for CTD spanning amino acid residues 76–119 and −3 for the region corresponding to model structure from residues 63 to 119. This indicated that the structural features of the YabA CTD model were of a quality comparable to high resolution structures (32). In addition, the YabA CTD (residues 76–119) sequence has 44% similarity and 35% identity with profilin, and accordingly the YabA model exhibited two short alpha-helices (Figure 3A, α2 and α3). Structural alignment with EEA1 revealed 12% identity and 19% similarity with YabA CTD. Similar to EEA1, the YabA zinc finger residues H95 and C110 (Figure 3A, upper case residues) and C97 and C112 (Figure 3A, lower case residues) were, respectively, inaccessible and accessible to solvent (Figure 3A, orange dots). These results stress the structural relevance of the two templates used here to build the YabA CTD model. The 3D model consists of two short helices (α2 and α3) followed by zinc binding loops (Figure 3B). Interestingly, the model positions the residues of the HCCC in a fold similar to the short zinc-binding loops found in other proteins (47).

Next, the model was assessed using knowledge of surface exposed residues. A previous functional dissection of YabA identified single residue changes that selectively disrupted interactions with one partner protein, while maintaining interactions with its other protein partners (15). In view of the selective effects of these LOI mutations, the substituted residues are expected to be exposed on the surface where their mutation is unlikely to affect the overall structure and stability of the protein. The sites of the LOI residue substitutions were mapped onto the YabA model (Figure 3C). The residues important for interaction with DnaA and DnaN were found to be surface exposed, defining non-overlapping interacting surfaces on the C-terminal zinc-binding domain. Residues Y83, N85 and L86, important for interaction with DnaA, are clustered on the helical side of the CTD surface when viewed as in Figure 3C while residues V99, H100 and L110 involved in interaction with DnaN are grouped predominantly on a surface formed by protein loops. This mapping of residues important for interaction with DnaA and DnaN to the surface of the YabA CTD provides an important validation of our *in silico* structural model.

### Low-resolution structure of the YabA tetramer

**Figure 4.**
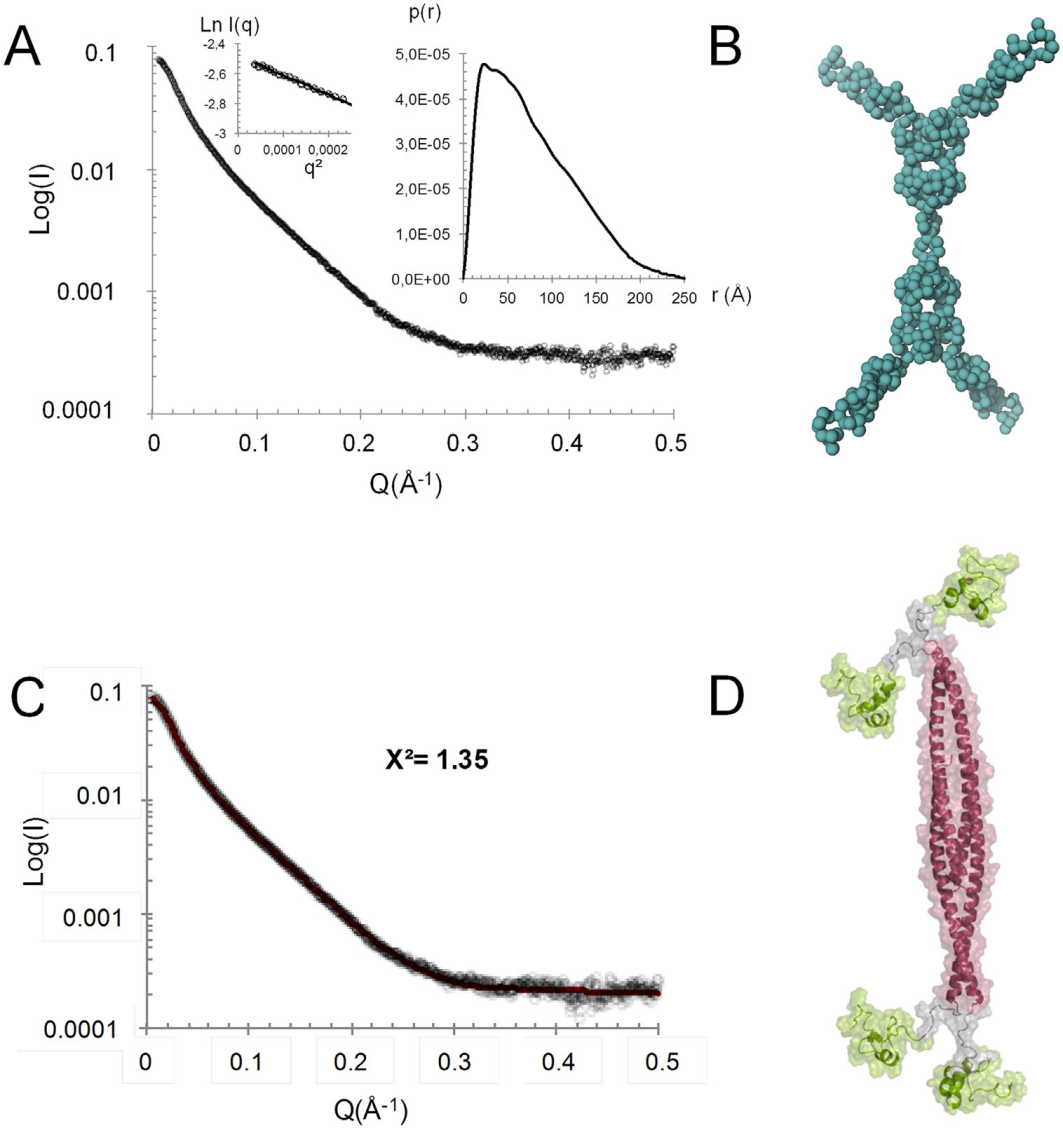
(**A**) Averaged scattering curve of the YabA protein coming from frames recorded on the main peak from SEC. The values of the radius of gyration Rg and the maximum dimension, Dmax, of the scattering particle are calculated from Guinier extrapolation (first box image) and autocorrelation function p(r) determination (second box image). (**B**) Bead model of YabA compatible with the SAXS data. (**C**) Averaged scattering curve of the YabA protein and the fitting curve calculated and superimposed with CRYSOL, coming from the model produced by Dadimodo under SAXS constraint. The logarithm of scattering intensity I is displayed as a function of the momentum transfer q = 4πsinθ/λ, where 2θ is the scattering angle and λ = 1.033 Å is the wavelength. (**D**) YabA structure model.

To gain insight into the overall architecture of the full-length tetrameric YabA, we used SAXS. YabA eluted from the online HPLC as a single peak and SAXS data indicated a radius of gyration (R_g_) of 63.2 Å and a maximum dimension (D_max_) of 250 Å (Figure 4A). These values are very high for a low molecular weight protein, even for a tetramer. The presence of a flexible region is suggested by the shape of the Kratky plot which displays a flattened bell shape at low values of s^2^ corresponding to the folded part of the protein, followed by a continuous rise at the higher angle sections (48) (Supplementary Figure S3A). Such dual behavior is indicative of flexibility in multidomain proteins (49). An extended YabA structure is also strongly suggested by the autocorrelation function with a shape characteristic of an elongated object (Figure 4A). In the crystal structure of the NTD, the C-termini of chains A and B’ (residues 62) are separated by ∼130 Å from the C-termini of chains A’ and B. The CTD model encompassing residues 76–119 is of around 25 Å at its maximum radius. Coupled with the observation from the SEC-MALS experiments that YabA^70–119^ is monomeric, these data suggest that residues 63–75 may act as an extended flexible linker between the NTD and the CTD. This is also supported by the fact that residues 60–80 are poorly conserved in YabA sequences (Supplementary Figure S1).

Using the scattering curve, a low-resolution structure of YabA was calculated by *ab initio* modeling with the program GASBOR without imposing symmetry constraints. In this case, we obtained a set of very different shapes (data not shown). To limit the range of possibilities, internal P_22_ symmetry was imposed, giving a shape composed of a cylindrical core with pairs of elongated arms attached to each end (Figure 4B). To gain better insights into the structure of the tetramer of YabA, we combined the crystal structure of the YabA NTD tetramer, with the CTD structure obtained by molecular modeling. Each N-terminal helix was connected to its cognate CTD domain by a linker composed of 14 residues (62–76). Modeling was performed with DADIMODO which allows a flexible linker region to be defined in the model while maintaining structural constraints imposed by molecular modeling processes and the SAXS data (see Materials and Methods, (40)). The model produced by DADIMODO fits the experimental scattering pattern (Figure 4C) and exhibits the same global organization as that obtained by *ab initio* modeling (Figure 4B and D). Individual runs revealed different solutions with variable positions of the CTDs, suggesting an intrinsic flexibility of the linker (Supplementary Figure S3B). Nevertheless, the *ab initio* and structure-guided modeling approaches converge to a common architecture for YabA: a core of four NTD α-helices each of which is connected to a globular CTD by a linker region in an extended conformation. The SAXS data and modeling experiments suggest that the linker confers a high degree of flexibility, leading to a multiplicity of conformations of the individual CTDs relative to the tetrameric core bundle Supplementary Figure S3B).

### Mutually exclusive interactions of DnaA and DnaN to the YabA C-terminal moiety

Previous studies revealed that YabA forms heterocomplexes with DnaA and DnaN *in vivo* (15,16). YabA was also shown to bridge DnaA and DnaN in a yeast three-hybrid assay (18). It was further shown that YabA LOI-mutations disrupting interactions with DnaA or with DnaN mapped to the CTD (15). Our structural and biophysical data established that the isolated CTD is monomeric, suggesting that these domains may function as independent binding sites in the YabA tetramer. We tested this assumption by determining whether YabA^62–119^, comprising the CTD globular domain together with the linker, was sufficient for interaction with DnaA or with DnaN in a yeast two-hybrid assay (Supplementary Figure S4A). We found that indeed, YabA^62–119^ was able to support interaction with either partner, implying that the NTD's quaternary structure is dispensable. This scheme raised the important question of whether a single CTD monomer could form simultaneous interactions with both DnaA and DnaN or whether the interactions with the two proteins are mutually exclusive. To answer this question, we performed a yeast 3HB assay in which a constitutively expressed YabA^62–119^ was tested for its ability to trigger interacting phenotypes by bridging DnaA and DnaN. We found that in contrast to the full length YabA, YabA^62–119^ was not able to bind simultaneously to DnaA and to DnaN whether they are fused with to the BD or the AD domains of Gal4. This observation sheds new light on the mode of binding of YabA to its partners. We conclude that DnaA and DnaN are able to bind concurrently to a YabA tetramer but not to a single CTD.

### Structural and functional determinants underlying the binding of YabA to DnaA and DnaN

Next, the model of the YabA CTD was used to predict additional residues involved in interactions with the partner proteins DnaA and DnaN. The predictions were based on residue surface accessibility calculations, sequence conservation analysis, comparison with templates and previous work (15). Candidate residues were targeted for scanning mutagenesis and the interaction profiles of the corresponding YabA mutants were determined experimentally in a yeast two-hybrid assay, similar to that previously used to identify LOI mutations (15).

**Figure 5.**
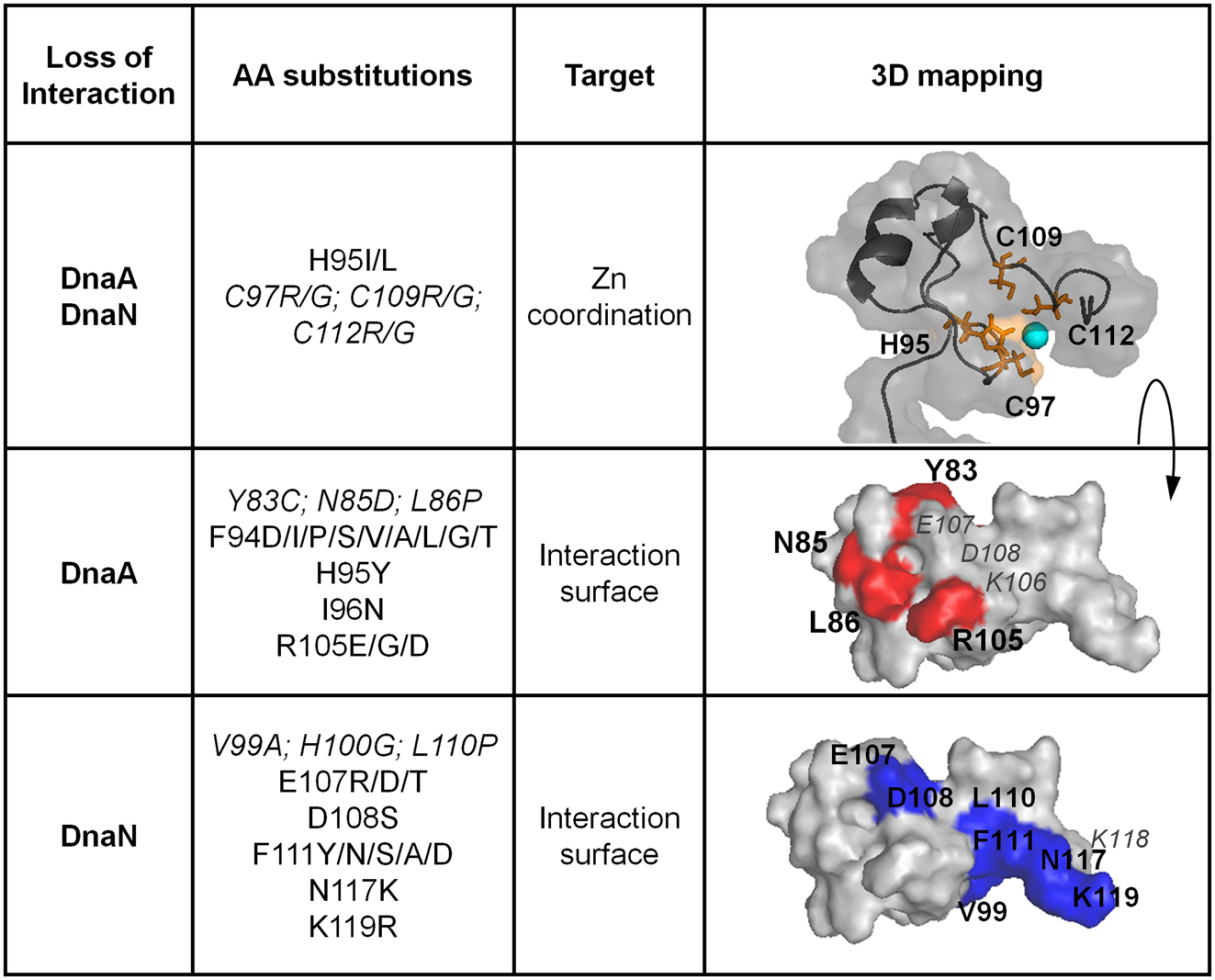
Surface representation of YabA showing key residues involved in interactions with partner proteins. Mapping of mutational data onto the structural model with interacting surfaces specified by color; DnaA (Blue) DnaN (red). Previous data is indicated in italic. Zn is illustrated by a cyan sphere. Residues with no interacting phenotypes are labeled in gray.

Previous work showed the importance of residues C97, C109 and C112 of the HCCC motif for interactions with both DnaA and DnaN (15). To experimentally investigate the role of the H95 of this motif, we tested the effects of targeted substitutions on the capacity of YabA to bind to its different partners. Replacement of H95 by I or L resulted in loss of interaction with both DnaA and DnaN while YabA–YabA self-interactions were preserved (Figure 5, Supplementary Table S3 and Figure S5). Similar effects were observed previously upon mutation of the conserved cysteine residues (15). This suggests that substitution of the polar H95 by hydrophobic residues results in structural defects that are confined to the CTD. Curiously, a H95G substitution did not exhibit an interaction defect in our yeast two hybrid assay (Supplementary Table S3). However this mutant, like the previously examined C97A mutant, exhibited a propensity to aggregate during expression in *E. coli* cells (data not shown). This is consistent with reduced stability caused by loss of Zn binding, although the direct participation of H95 in Zn coordination is not yet proved. Substitutions of the neighboring residues, F94 and I96 also impeded interaction with DnaA but had only moderate effects on interaction with DnaN (Supplementary Table S3). The hydrophobic residue F94, is highly conserved as a F or Y among YabA homologs and is buried in our model (Supplementary Figure S1). The F94Y mutation in YabA did not affect protein interactions. In contrast, substitution of F94 by a variety of polar and aliphatic residues led to specific defects in DnaA interactions (Figure 5, Supplementary Table S3 and Figure S5). These results suggest that the H95 flanking residues, F94 and I96, are also important for the folding of the CTD. Together these results support the importance of a structured domain in which Zn-coordination might play a role.

To further delineate the DnaA binding surface, we targeted residues R105 and K106, as these neighbor the surface-exposed residues N85 and L86 (previously shown to be important for DnaA binding). YabA mutants R105E, R105D and R105G lost the ability to bind to DnaA. However substitution of K106 by a range of amino acid residues had no effect on DnaA binding (Figure 5, Supplementary Table S3 and Figure S5). This suggests that R105 but not K106 is part of the DnaA binding site (Figure 5 and Supplementary Table S3). YabA substitutions of the neighboring residues E107and D108 did not exhibit LOI phenotypes for DnaA, indicating these residues are not part of the DnaA binding surface. Instead these substitutions produced a DnaN LOI phenotype. This suggests that the two interacting surfaces may be overlapping.

To further delineate the DnaN interaction surface, mutagenesis of F111, N117 and the terminal K118 and K119 residues was performed. Whilst, the substitution of K118 by various amino-acid residues had no effect on protein interactions, mutations targeting residues F111, N117 and K119 all resulted in DnaN LOI mutants (Figure 5, Supplementary Table S3 and Figure S5). The location of residues F111, N117 and K119 in the CTD model are consistent with their role in DnaN binding as they are predominantly surface exposed. Moreover, these residues are adjacent to V99 and L110 previously found to be important for DnaN binding. Taken together, these data clearly delineate the interaction determinants of DnaA and DnaN on two partially overlapping surfaces on the YabA CTD.

### Effect of loss-of-interaction mutations on sub-cellular localization in *B. subtilis* cells

**Figure 6.**
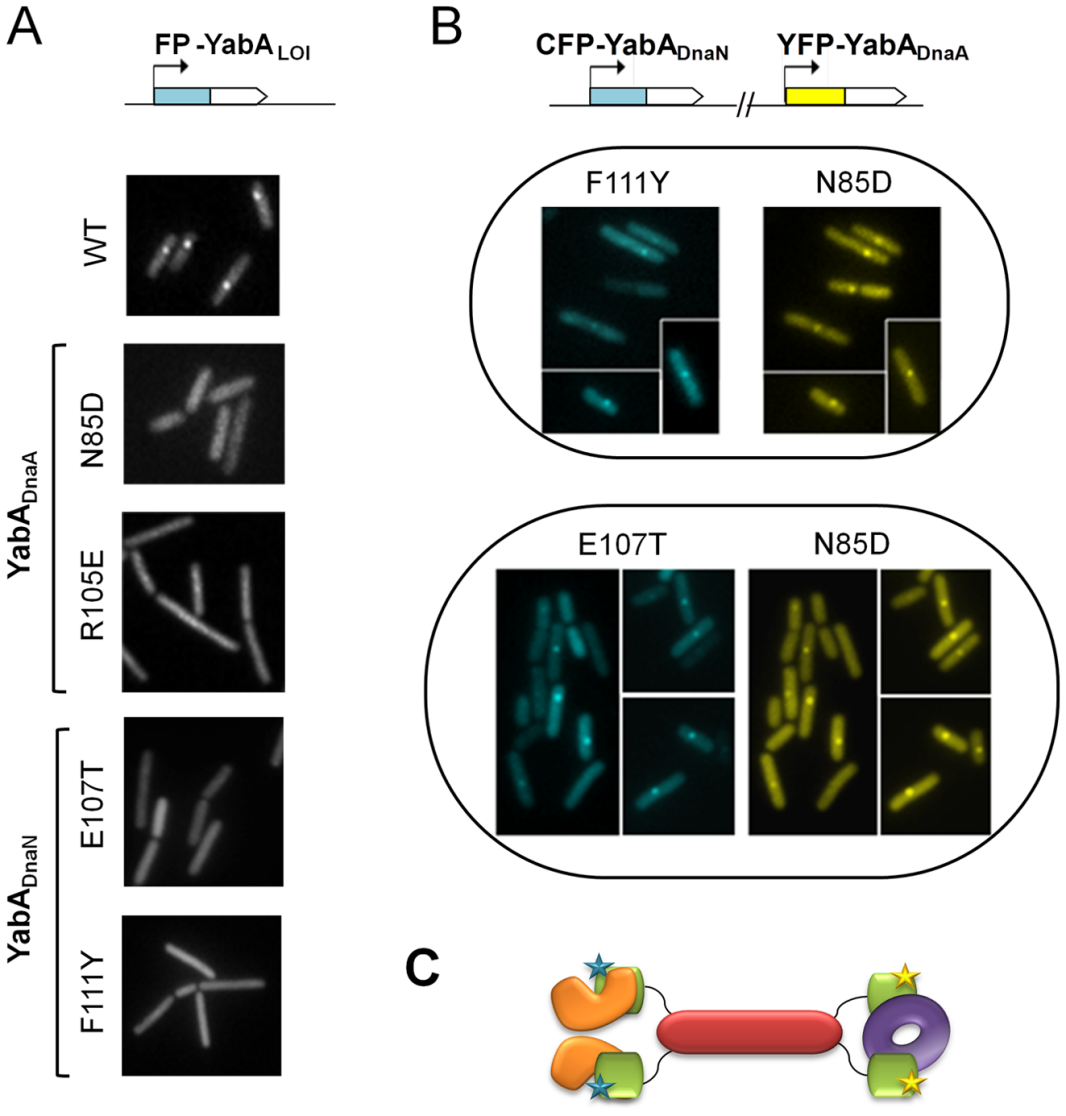
Cellular localization of YabA-WT and -LOI mutants by fluorescence monitoring of signals from CFP- (cyan) or YFP- (yellow) YabA fusions in living cells. (**A**) Impaired localization of YabA LOI mutants in DnaA (N85D and R105E) and in DnaN (E107T and F111Y) compared to YabA-WT. (**B**) Restoration of localization of YabA_DnaN_-LOI mutants by cross-complementation with a YabA-_DnaA_-LOI mutant. The co-expression in the same cell of the YabA-_DnaN_ mutants F111Y or E107T fused to CFP (blue) with a YabA-_DnaA_ N85D fused to YFP (yellow) restored the ability of each YabA mutant derivative to localize as a focus at the cell centre. The co-localization of foci indicates that they are part of a DnaA/YabA/DnaN complex, where the YabA tetramer is potentially composed of a mixture of YabA-_DnaN_ (proficient for binding to DnaA) and YabA-_DnaA_ (proficient for binding to DnaN) mutants. (**C**) Model illustrating cross complementation between YabA-_DnaA_ (CTDs in green with yellow stars) and YabA_DnaN_ (CTDs in green with blue stars) for the binding and the cellular localization of DnaN (purple doughnut) and DnaA (orange). The YabA NTD's tetrameric core is represented in Indian red.

YabA forms a heterocomplex with DnaA and DnaN which co-localizes with the replication machinery for most of the cell cycle (15,17). To validate our structure predictions, we investigated the subcellular localization of several YabA_DnaA_ and YabA_DnaN_-LOI mutants in living cells by transferring the corresponding point mutations into a *yabA* gene fused in-frame to *cfp or yfp*. Examination of the cellular protein levels revealed that the YabA mutant derivatives were present at similar or higher levels compare to the wild-type (Supplementary Figure S6). Wild-type YabA localizes as one or two foci per cell, with single foci displaying a midcell localization (Figure 6, (15,17)). LOI with either DnaA or DnaN is associated with both loss of YabA localization and loss of initiation control (15). Here, we observed that F94S and R105E substitutions in a CFP-YabA fusion give a fluorescence signal dispersed throughout the cell, similar to that of the YFP-YabA-N85D mutant identified in a previous functional study (15) (Figure 6A and Supplementary Figure S7). Since F94S and R105E substitutions specifically impaired the binding of YabA to DnaA (but not DnaN) in the yeast two-hybrid assays, we conclude that these two residues contribute to the integrity of the YabA/DnaA interface together with residues N85 and L86 identified formerly. The R105E and F94S substitutions reduce but do not completely abolish foci formation since patches of increased fluorescence intensity can still be discerned in parts of the cells, in agreement with the observation of Goranov *et al*. that YabA foci can be detected in cells deficient in DnaA-mediated initiation of chromosomal replication (50). Thus, while these two substitutions cause significant dispersal of YabA throughout the cell, residual YabA co-localization with the replication machinery could still be observed. Examination of CFP-YabA fusions carrying the DnaN-LOI substitutions E107T, E107R, D108S or F111Y, revealed that foci formation was completely abolished as observed previously for DnaN-LOI substitutions of V99 and L110 (Figure 6A, Supplementary Figure S7). These observations support the assignment of these residues to the DnaN interacting surface. Overall, the data are consistent with the predictions of the structural model of YabA.

When co-expressed in the cell, DnaA- and DnaN-interaction deficient mutants of YabA can cross-complement and restore YabA cellular localization and function (15). Here we tested the capacity of the DnaA-LOI YabA mutant N85D of YabA, to restore the localization of the DnaN-LOI mutants (F111Y and E107T). The CFP-YabA-F111Y (or E107T) and the YFP-YabA-N85D fusion proteins are primarily dispersed in the cell when expressed alone (Figure 6A), but re-localized as sharp mid-cell foci containing both CFP and YFP fluorescence when co-expressed in the same cell (Figure 6B). This functional complementation of DnaA and DnaN LOI mutations indicates that the mutant YabA proteins are properly folded in the cell, and that the interaction deficiency of each mutant is complemented by the other in a functional heterocomplex that localizes correctly at mid-cell (Figure 6C). These results fully support the assignment of residues E107 and F111 to the DnaN-binding surface thus lending further experimental support to the structural model. These observations suggest that the CTD harbors the determinants for association with the replication machinery while the N-terminal domains account for the quaternary structure of YabA perhaps allowing additional levels of regulation of its activity.

## DISCUSSION

In *B. subtilis*, YabA is proposed to exert its negative control of replication initiation by concurrent mechanisms at work at the replication origin and at the replication factory. YabA inhibits the formation of a DnaA-nucleoprotein structure at *oriC* by binding to free DnaA thus reducing its availability in the cell (13,14). YabA also tethers DnaA with the β-clamp DnaN in a heterocomplex, which co-localizes with the replication factory during most of the replication cycle (15–16,18). Thus, YabA acts to prevent premature re-initiation by trapping DnaA in a DnaN-independent or a DnaN-dependent manner, respectively. The question of how YabA coordinates its actions to allow the transition from initiation to elongation of replication remains obscure and a better understanding of the structural organization and dynamic character of the complexes it forms with DnaA and DnaN is needed.

Our study establishes that YabA assembles as a head-to-head dimer of dimers in solution, through coiled-coil interactions of its helical NTDs. The C-terminal portions of YabA fold as independent domains linked to their cognate NTDs by a short flexible region. YabA CTD is a zinc-associated domain that mediates binding to DnaA and to DnaN. Analysis of the surfaces involved in binding to DnaA and DnaN revealed they are partly overlapping, thus ruling out the possibility that the two proteins bind concomitantly to the same CTD. As a result, ternary interactions of YabA, DnaA and DnaN are observed only if the full length YabA, able to self-assemble through its NTD, is expressed in the yeast 3HB assay. Importantly, the capacity of YabA_DnaA_ and YabA_DnaN_ LOI mutants to complement each other's localization defects and restore foci at the replication factory when co-expressed in *B. subtilis* cells provides strong evidence to substantiate the hypothesis that DnaA, YabA and DnaN can be part of the same hetero complex. Altogether our results suggest strongly that DnaA and DnaN associate with separate CTDs of the YabA tetramer.

Examination of residues important for interaction with DnaA and DnaN showed that they clustered in partially overlapping patches. Protein–protein interfaces are generally composed of different types of residues that contribute unevenly to the binding free energy. Scanning mutagenesis experiments allow three classes of residues to be defined according to their contribution to protein–protein complex formation (51). Interfaces are composed of (i) core residues, which become extensively buried upon complex formation and whose mutation leads to drastic lowering of complex stability. These residues have been defined as ‘hot spots’ and they are typically highly evolutionary conserved (52); (ii) rim residues which surround the core, sheltering the latter from the solvent, and whose mutation generally tends to have modest effects on complex stability (52,53) and (iii) residues which do not contribute directly to the interface but which play an important role in defining its structural integrity. Our refinement of the DnaA interacting surface revealed novel conserved residues that fall in two of these categories. Firstly, a three amino-acid residue cluster FHI at positions 94–96, encompassing the conserved H95 of the HCCC motif, was found to be important for interaction with DnaA. We propose that this cluster is most likely involved in the stabilization of the zinc-containing fold and contributes to the structural integrity of the binding surface. Secondly, we found that the surface-exposed and positively charged residue R105, is critical for DnaA binding to YabA. R105 is strictly conserved among the YabA-family of protein. We propose that R105 constitutes a binding hotspot, in agreement with the observation that arginines are frequently represented in hot spots (52). Together with N85 and L86, R105 is part of an interacting patch highly conserved in the YabA family of proteins.

YabA exerts part of its regulatory mechanism by preventing DnaA oligomerization at *oriC* (13,14). DnaA mutant derivatives involving residues H162 and A163 were found to be insensitive to YabA control (14). These two residues are located in domain III of DnaA, which is important variously for binding to ssDNA, for ATP hydrolysis and for DnaA oligomerization. In the three dimensional structure of DnaA, H162 and A163 are in close proximity to residue F120 which is known to be important for interactions with YabA and DnaD (1,14,54). Whether these residues play a role in binding to YabA directly or indirectly remains to be established. The available structural data on YabA and DnaA from *B. subtilis* is insufficiently detailed to allow precise inferences on the YabA:DnaA interface to be drawn. However, analysis of the of a crosslinked YabA:DnaA complexes prepared by Schoefield *et* *al*., revealed the dominance of a 1:1 complex stochiometry with a low proportion of 2:1 stochiometry (14). We can anticipate that YabA could bind to one DnaA unit per YabA-CTD domain.

Proteins interacting with the bacterial sliding clamp usually possess a short linear binding consensus signature QLxLF (19). Remarkably, no sequence related to this consensus is present in YabA (15). Although hydrophobic residues were identified, the composition and spatial arrangement of the surface-exposed residues involved in binding to DnaN suggest a different mode of interaction for YabA. Among the YabA_DnaN_ surface residues only one, F111, is highly conserved among the YabA family of proteins. Residues at this position are strictly hydrophobic, represented by F (82%) or L (18%) (Supplementary Table S1) suggesting this might contribute substantially to the free energy of binding. In YabA, further amino-acid residues including two hydrophobic residues (V99 and L110), two acidic residues (E107 and D108) and a positively charged residue at the C-terminus (K119) have been found to contribute to binding to DnaN. These less conserved residues might be important for tuning the affinity and/or the specificity of the interaction. In summary, our results point to a YabA β-binding mode that differs from other β-binding proteins such as the replicative DNA polymerase and presumably requires a different interacting surface. This result is in agreement with the DnaN-dependent localization of YabA at the replication machinery for most of the bacterial cell cycle (15,17).

The structural characterization of YabA reveals a hub architecture. The structure of YabA is reminiscent of that of the FtsZ associated protein ZapA which is recruited to the divisome machinery to stabilize the Z-ring during bacterial cytokinesis (55–57). ZapA assembles into an elongated anti-parallel tetramer, in which two subunits associate via their coiled coil CTDs and two dimers then associate into a tetramer. At both ends of the tetrameric helical bundle, globular NTDs comprise the determinants for binding to FtsZ (56). An important aspect of such homo-oligomeric proteins is their ability to contact and connect different protein partners. In *E. coli*, ZapA recruits ZapB to the Z-ring (58,59). In *B. subtilis*, ZapA interacts with different components of the division machinery including SepF and EzrA (60). The architecture of YabA is also similar to the eukaryotic protein Geminin, which downregulates DNA replication through the timely inhibition of the licensing factor Cdt1 (61,62). Geminin forms a parallel coiled-coil homodimer that binds to Cdt1. It has been proposed that regulation of initiation is exerted though a switch in quaternary structure from a permissive Geminin_2_/Cdt1 heterotrimer to an inhibitory Geminin_4_/Cdt1_2_ heterohexamer (61). Auxiliary basic and acidic residues in Cdt1 are involved in binding to PCNA, a parallel which may be extended to the recruitment of DnaN by YabA. Although these structural resemblances between proteins regulating replication in bacterial and eukaryotic cells do not imply similar regulatory mechanisms, they suggest that the evolutionary constraints on preventing re-replication have led to targeted interactions with key factors involved in both initiation and elongation.

Thus YabA, Geminin and ZapA represent examples of the exploitation of a coiled-coil helical core as a protein hub allowing the assembly and disassembly of protein complexes in a dynamic way. The structural organization of YabA offers a simple explanation for how it forms heterocomplexes with DnaA and DnaN. The flexible C-terminal moieties provide YabA with the dynamic capacity to interact (i) with more than one DnaA molecule, preventing the helical assembly of the latter at *oriC*, or (ii) after initiation with both DnaA and DnaN at the replication machinery. In the latter case, we anticipate that the two CTDs (say the AB’ pair) at one end of the YabA tetramer could contact symmetrical binding surfaces on the ring-shaped DnaN dimer, with up to two DnaA monomers, interacting with the CTD pair (A'B) pair at the opposite end of the complex as depicted in Figure 6C. Future studies are required to gain insights into the architecture and stoichiometry of the different complexes YabA forms with its partners. Nevertheless, our experimentally informed structural model of the YabA tetramer sheds light on how YabA can mediate multiple interactions, and lays the foundation for further understanding of how YabA coordinates its actions to down-regulate replication initiation.

## Supplementary Material

SUPPLEMENTARY DATA
