# Supplementary material for: Tetramerization and interdomain flexibility of the replication initiation controller YabA enables simultaneous binding to multiple partners
Source: Nucleic Acids Res. 2015 Nov 28;44(1):449–63. doi: 10.1093/nar/gkv1318 (PMC4705661; doi:10.1093/nar/gkv1318)
Supplement: SUPPLEMENTARY DATA [file supp_44_1_449__index.html]

Tetramerization and interdomain flexibility of the replication initiation controller YabA enables simultaneous binding to multiple partners — Tetramerization and interdomain flexibility of the replication initiation controller YabA enables simultaneous binding to multiple partners — SUPPLEMENTARY DATA 

# Tetramerization and interdomain flexibility of the replication initiation controller YabA enables simultaneous binding to multiple partners

## SUPPLEMENTARY DATA

- SUPPLEMENTARY DATA
- SUPPLEMENTARY DATA
