# Supplementary material for: Tetramerization and interdomain flexibility of the replication initiation controller YabA enables simultaneous binding to multiple partners
Source: Nucleic Acids Res. 2015 Nov 28;44(1):449–63. doi: 10.1093/nar/gkv1318 (PMC4705661; doi:10.1093/nar/gkv1318)
Supplement: SUPPLEMENTARY DATA [file supp_gkv1318_nar-02449-v-2015-File009.pdf]

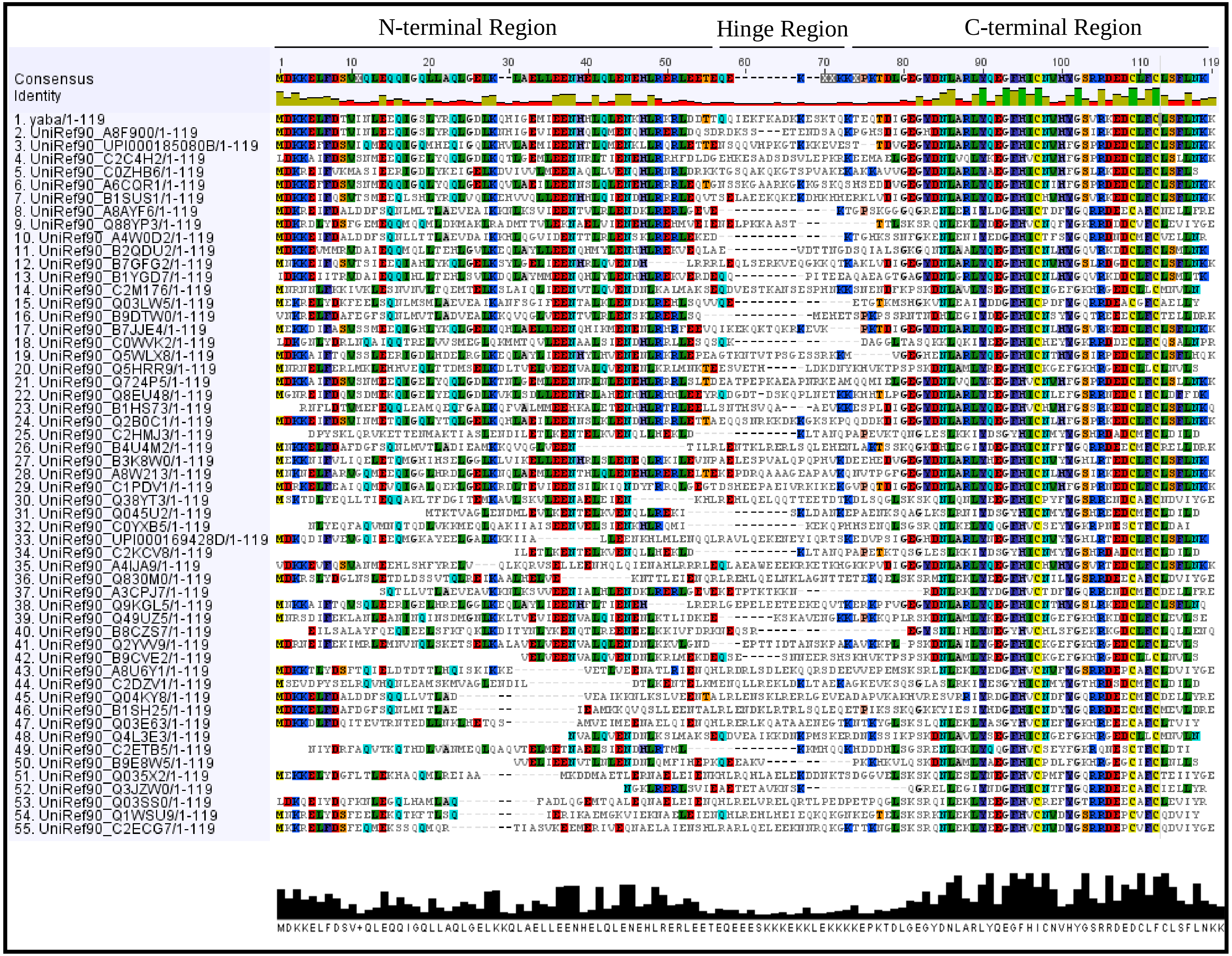

Figure S1: YabA multiple sequence alignment.
The algorithm Jnet implemented through Jalview version 2 software (1) was used to search for similarities to the YabA sequence in UniRef90 (2). An alignment was constructed with PSI-BLAST (3). In the alignment, sequence names are composed of UniRef90 + SwissProt identifier + protein length. A histogram of amino acid residue conservation in the consensus sequence is shown below.

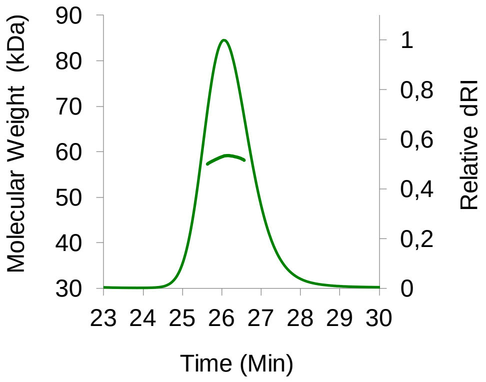

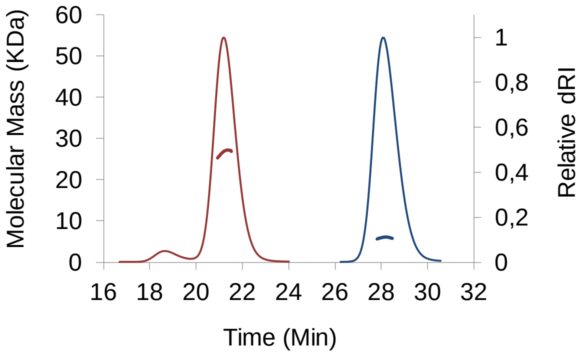

Figure S2

Molecular weight analysis of YabA and the NTD and CTD fragments (green, red and blue curves, respectively) by Size Exclusion Chromatography -Multi-Angle Laser Light Scattering (SEC-MALLS). The continuous lines trace the differential refractive index of the eluate from a Superdex column as a function of time. The thicker lines represent the weight average molecular weight of the species in the eluate, calculated from refractive index and light-scattering measurements. Here the chromatograms for YabA$^{1\text{-}58}$ (red) and YabA$^{70\text{-}119}$ (blue) are overlaid. The experimentally determined molecular masses are 58.7, 27.7 and 6.2 kDa respectively.

A

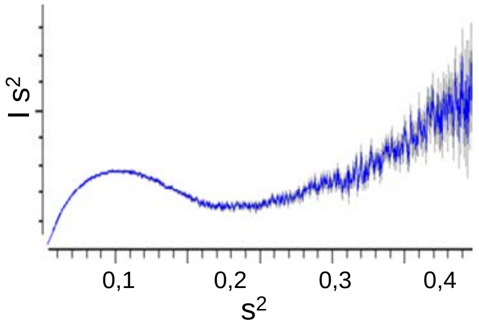

B

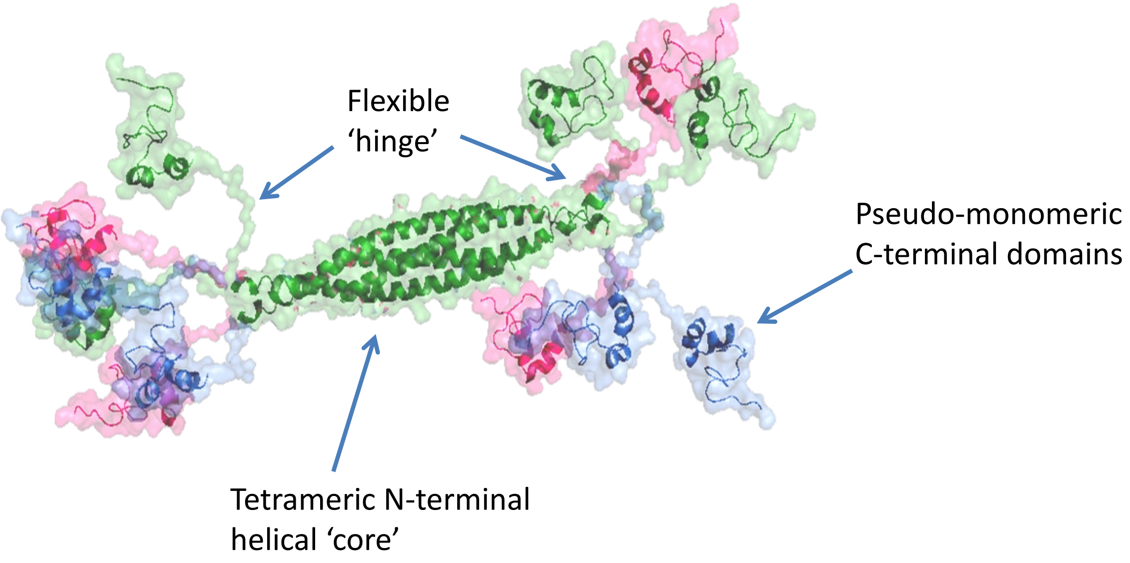

Figure S3

Flexibility of YabA structure; A) Kratky Plot of YabA scattering data. B) Representative models resulting from rigid body modeling to the SAXS data. Here is represented three models suggesting that the ordered NTD and CTD are joined by a linker of structurally dissimilar states, featuring huge flexibility. The NTD is fixed (green) and each CTD within a YabA tetramer is pictured as a different color.

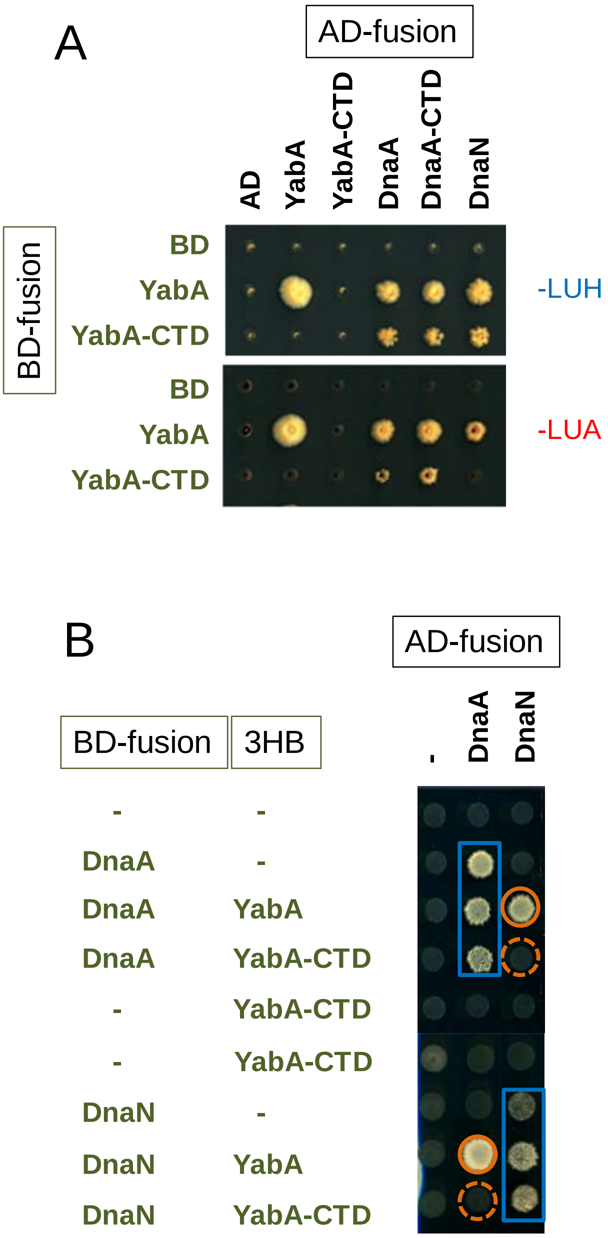

Figure S4

Analysis of YabA C-terminal requirement for interaction by yeast 2HB and 3HB. A) y2HB assay: The BD and AD (rows) fusions are indicated. The binary interaction phenotypes correspond to the ability of the diploids cells to growth onto SD-LUH and SD-LUA selective media. B) 3HB assay: BD- fusion proteins are co-expressed with another protein partner (3HB), as indicated. Control vectors without insert are indicated by a dash. A ternary interaction is revealed by the ability of diploids to express interacting phenotypes (ie- growth onto SD-LUWH) conditionally to the presence of a third partner (indicated by oranges circles). Dashed circles indicate absence of ternary interaction when YabA-CDT is expressed instead of YabA full size. In this assay, binary interactions are revealed by the apparition of the interacting phenotype independently of the presence of a third partner (such as DnaA/DnaA, blue rectangle). A particular note to DnaN that self-interacts weakly in the y2HB, probably caused by the steric hindrance of Gal4 domains at the N-terminal part of DnaN, involved in the head to tail binding of two momoners into a circlular doughnut.

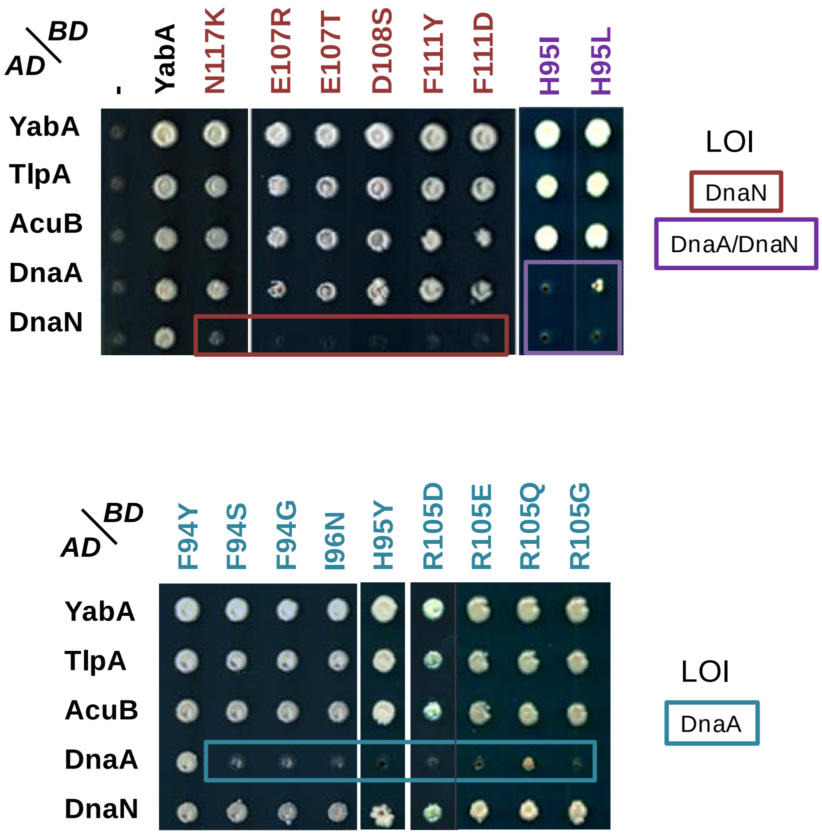

Figure S5

Analysis of interacting phenotypes of YabA-LOI (Loss of Interaction) mutants by yeast 2HB. Constructions were carried out as described in Material and Methods. BD- and AD- fusions were expressed in compatible haploid cells and combination pairs are co-expressed in the diploid cells after mating as described in Fig. S4. YabA wild type and LOI mutant derivatives were assayed for self interaction, as well as interaction with all the four partners (DnaA, DnaN,TlpA and AcuB) identified in the yeast two hybrid (4). $\text{YabA}_{\text{DnaA}}$ (or $\text{YabA}_{\text{DnaN)}}$ LOI mutations are characterized by the loss of interaction phenotypes when co-expressed with DnaA (or DnaN) while still exhibiting self interaction as well as interaction phenotypes when expressed with the other partners, incuding TlpA and AcuB.

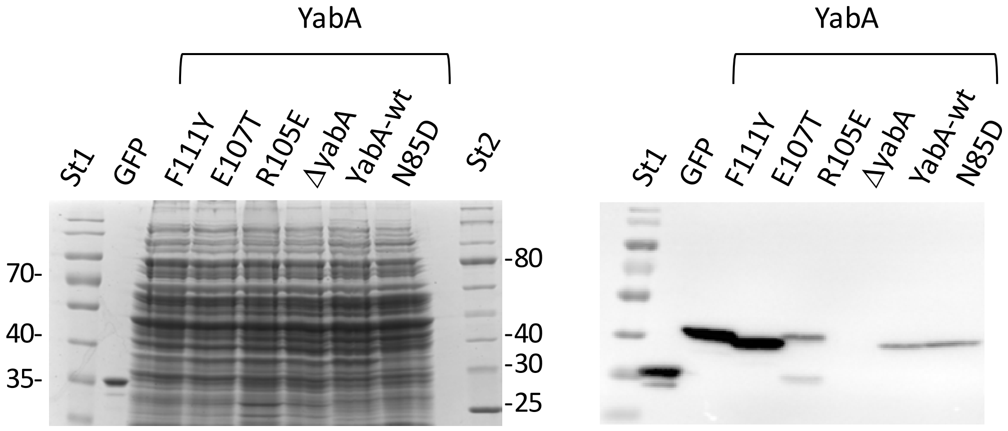

Figure S6: Immunodetection of the GFP-YabA fusions: Expression of YabA fusion proteins was confirmed by western blot, using Living colors Monoclonal anti-GFP Antibody (JL-8) (from Takara) as primary antibody revealed by goat anti-mouse antibody HRP conjugate, from equivalent amounts of total cell lysates *of B. subtilis* strains expressing the YFP or CFP-YabA wt and mutant derivatives. Purified GFP protein is used as a control. Left: Coomassie-stained and right: Western blot. The difference of signal intensity between CFP-YabA derivatives (F111Y and E107T) and YFP-YabA derivatives (WT, N85D) observed here, can be attributed either to a higher expression of the CFP-derivatives or to a differential sensitivity of the antibodies toward the CFP or YFP variants. Slight but noticeable proteolysis is observed in the CFP-YabA-R105E that gives rise to the detection of free CFP.

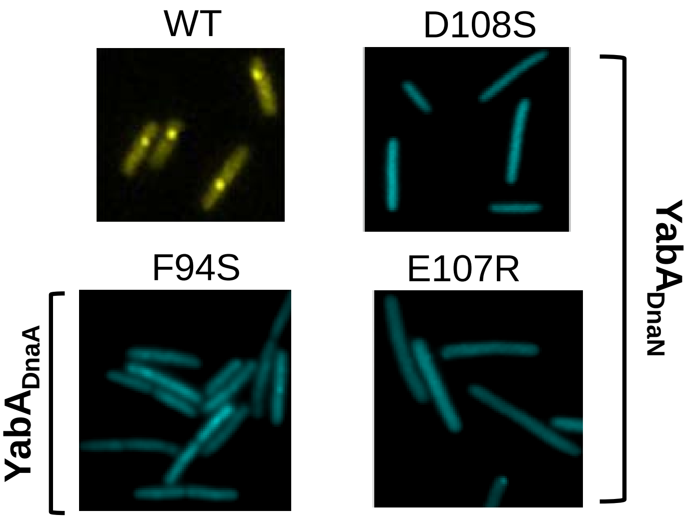

Figure S7

Cellular localisation of YabA_LOI mutants; additional information.

Impaired localization of GFP-YabA LOI mutants in DnaA (F94S) and in DnaN (E107R, and D108S) compare to YabA-WT (see legend Fig. 6).

References:

1. Waterhouse, A.M., Procter, J.B., Martin, D.M., Clamp, M. and Barton, G.J. (2009) Jalview Version 2--a multiple sequence alignment editor and analysis workbench. *Bioinformatics (Oxford, England)*, **25**, 1189-1191.
2. Suzek, B.E., Huang, H., McGarvey, P., Mazumder, R. and Wu, C.H. (2007) UniRef: comprehensive and non-redundant UniProt reference clusters. *Bioinformatics (Oxford, England)*, **23**, 1282-1288.
3. Altschul, S.F., Madden, T.L., Schaffer, A.A., Zhang, J., Zhang, Z., Miller, W. and Lipman, D.J. (1997) Gapped BLAST and PSI-BLAST: a new generation of protein database search programs. *Nucleic acids research*, **25**, 3389-3402.
4. Noirot-Gros, M.F., Dervyn, E., Wu, L.J., Mervelet, P., Errington, J., Ehrlich, S.D. and Noirot, P. (2002) An expanded view of bacterial DNA replication. *Proc Natl Acad Sci U S A*, **99**, 8342-8347.
