# Supplementary material for: Tetramerization and interdomain flexibility of the replication initiation controller YabA enables simultaneous binding to multiple partners
Source: Nucleic Acids Res. 2015 Nov 28;44(1):449–63. doi: 10.1093/nar/gkv1318 (PMC4705661; doi:10.1093/nar/gkv1318)
Supplement: SUPPLEMENTARY DATA [file supp_gkv1318_nar-02449-v-2015-File010.pdf]

Table S1 - Putative YabA proteins currently described as hypothetical proteins or proteins with unknown function.

Protein Description	Organism	Accession Number	Score, Query coverage, E-value
protein of unknown function DUF972	Bacillus coagulans 36D1	ZP_04431360.1	68.5, 100%, 2e-10
hypothetical protein BSG1_10613	Bacillus sp. SG-1	ZP_01861149.1	67.3, 100%, 5e-10
protein of unknown function DUF972	Bacillus selenitireducens MLS10	YP_003698155.1	66.2, 100%, 1e-09
protein of unknown function DUF972	Bacillus cellulosilyticus DSM 2522	ZP_06363936.1	65.4, 97%, 2e-09
conserved hypothetical protein	Bacillus cereus AH1134	ZP_03233554.1	65.0, 100%, 3e-09
conserved hypothetical protein	Listeria monocytogenes str. 4b H7858	ZP_00230634.1	64.3, 100%, 5e-09
hypothetical protein lp_0706	Lactobacillus plantarum WCFS1	NP_784465.1	63.9, 97%, 6e-09
protein of unknown function DUF972	Geobacillus sp. WCH70	YP_002948237.1	63.9, 100%, 7e-09
hypothetical protein lse_0147	Listeria seeligeri serovar 1/2b str. SLCC3954	YP_003463390.1	63.5, 100%, 7e-09
protein of unknown function DUF972	Paenibacillus sp. JDR-2	YP_003008785.1	63.1, 97%, 9e-09
protein of unknown function DUF972	Geobacillus sp. Y412MC10	YP_003240136.1	63.1, 97%, 1e-08
hypothetical protein PTH_0065	Pelotomaculum thermopropionicum SI	YP_001210615.1	61.9, 100%, 2e-08
hypothetical protein Swol_0044	Syntrophomonas wolfei subsp. wolfei str. Goettingen	YP_752774.1	61.9, 97%, 2e-08
hypothetical conserved protein	Geobacillus kaustophilus HTA426	BAD74312.1	61.6, 97%, 3e-08
hypothetical protein CAT7_06883	Carnobacterium sp. AT7	ZP_02184374.1	61.6, 97%, 3e-08
hypothetical protein HM1_0756	Heliobacterium modesticaldum Ice1	YP_001679810.1	61.2, 100%, 4e-08
hypothetical protein GALLO_1751	Streptococcus gallolyticus UCN34	YP_003431166.1	61.2, 100%, 4e-08
hypothetical protein SDEG_0452	Streptococcus dysgalactiae subsp. equisimilis GGS_124	YP_002996168.1	61.2, 100%, 4e-08
hypothetical protein Exig_0027	Exiguobacterium sibiricum 255-15	YP_001812531.1	61.2, 97%, 4e-08
hypothetical protein STRINF_01993	Streptococcus infantarius subsp. infantarius ATCC BAA-102	ZP_02921109.1	61.2, 100%, 4e-08
hypothetical protein EF2760	Enterococcus faecalis V583	NP_816388.1	60.8, 97%, 5e-08
protein of unknown function DUF972	Bacillus tusciae DSM 2912	YP_003587970.1	60.8, 97% ,5e-08
hypothetical protein LAF_029	Lactobacillus fermentum IFO 3956	YP_001843106.1	60.4, 84%, 6e-08
protein of unknown function DUF972	Exiguobacterium sp. AT1b	YP_002886073.1	60.4, 97%, 7e-08
protein of unknown function DUF972	Thermincola sp. JR	YP_003638886.1	60.0, 97%, 9e-08
hypothetical protein LSA0343	Lactobacillus sakei subsp. sakei 23K	YP_394956.1	59.6, 97%, 1e-07
Protein of unknown function DUF972	Enterococcus faecium DO	ZP_00603925.1	58.9, 97%, 2e-07
conserved hypothetical	Streptococcus pyogenes	BAC64661.1	58.1, 97%, 3e-07

protein	SSI-1		
hypothetical protein Dred_0062	Desulfotomaculum reducens MI-1	YP_001111437.1	58.1, 97%, 3e-07
hypothetical protein CHY_0053	Carboxydothermus hydrogenoformans Z-2901	YP_358925.1	57.7, 100%, 5e-07
conserved hypothetical protein	Lactobacillus jensenii 269-3	ZP_04645668.1	57.7, 94%, 5e-07
protein of unknown function DUF972	Desulfotomaculum acetoxidans DSM 771	YP_003189689.1	57.3, 100%, 5e-07
hypothetical protein GCWU000282_00875	Catonella morbi ATCC 51271	ZP_04449646.1	57.3, 94%, 5e-07
hypothetical protein Moth_0045	Moorella thermoacetica ATCC 39073	YP_428927.1	56.9, 100%, 6e-07
protein of unknown function DUF972	Syntrophothermus lipocalidus DSM 12680	YP_003701422.1	56.2, 94%, 1e-06
hypothetical protein DSY0124	Desulfitobacterium hafniense Y51	YP_516357.1	56.2, 97% 1e-06
protein of unknown function DUF972	Desulfitobacterium hafniense DCB-2	YP_002456568.1	56.2, 97%, 1e-06
hypothetical protein Hore_20340	Halothermothrix orenii H 168	YP_002509774.1	54.6, 100%, 3e-06
conserved hypothetical protein	Lactobacillus coleohominis 101-4-CHN	ZP_05553573.1	54.6, 97%, 3e-06
conserved hypothetical protein	Staphylococcus hominis SK119	ZP_04060918.1	54.6, 92%, 3e-06
hypothetical protein SAD30_0199	Staphylococcus aureus D30	ZP_06022830.1	54.6, 89%, 4e-06
protein of unknown function DUF972	Alicyclobacillus acidocaldarius LAA1	ZP_03493323.1	53.9, 97%, 6e-06
protein of unknown function DUF972	Lactobacillus reuteri 100-23	ZP_03074103.1	53.9, 94%, 6e-06
hypothetical protein Lreu_0333	Lactobacillus reuteri DSM 20016	YP_001270940.1	53.5, 94%, 7e-06
hypothetical protein MCCL_1926	Macrococcus caseolyticus JCSC5402	YP_002561329.1	50.4, 89%, 7e-05
hypothetical protein Sca_0127	Staphylococcus carnosus subsp. carnosus TM300	YP_002633225.1	50.0, 97%, 9e-05

Table S2- Detection of proteins with distant sequence or structural relationships with the C-terminal region of YabA using PHI-BLAST

**PHI-BLAST Pattern**[a]	**Non-redundant databank**			**Protein Data Bank (PDB)**			
	**Accession number**	**Query cover**	**E-value**	**PDB Code**	**Query cover**	**E-value**	**Zinc finger motif**
H-x-C-x(11)-C-x(2)-C	ABG65911.1	72 %	7e-08	2EA5-A	20 %	2e-06	RING finger domain C3HC4 (2 Zn)
	AAL25175.1	72 %	6e-07	2CON-A	21 %	2e-06	Zn-ribbon module C4
	XP_001319569.1	67 %	2e-04	2ECL-A	17 %	1e-04	Ring-box protein C6H2C4 (3 Zn)
F- H-x(1,4)-C-x(7,14)-C-x(2)-C	YP_002379624.1	73 %	2e-05	2G6Q-A	43 %	1e-18	PHD domain C4HC3 (2 Zn)
	AAI72933.1	73 %	1e-04	1WES-A	43 %	1e-18	PHD domain C4HC3 (2 Zn)
	AAI44321.1	73 %	1e-04	2KIJ-A	47 %	3e-16	No Zn finger motif
H-x(1,4)-C-x(7,14)-C-x(2)-C	YP_002379624.1	73 %	1e-05	1JOC-A	63 %	3e-21	FYVE domain C8 (2 Zn)
	AAI72933.1	73 %	3e-05	2K2C-A	50 %	4e-10	CHY, CTCHY zinc finger domain, Ring finger (6 Zn) CHC4H2C4, C2HC + C2HC3HC
	AAI44321.1	73 %	4e-05	2DKT-A	49 %	3e-09	Ring finger and CHY zinc finger domain (6 Zn) CHC4H2C4, C2HC + C2HC3HC
C-x(7,14)-C-x(2)-C	YP_002379624.1	73 %	2e-05	1JOC-A	63 %	2e-24	FYVE domain C8 (2 Zn)
	AAI72933.1	73 %	7e-05	1HYI-A	28 %	2e-05	FYVE domain C8 (2 Zn)
	AAI44321.1	73 %	9e-05	2QA7-A	73 %	0.037	No Zn finger motif

[a] Patterns are described using the one-letter amino acid code. ‘x’ represents any amino acid. The numbers inside parenthesis represent the distance between two given amino acids

For the Non-redundant databank, only ABG65911.1 has a zinc finger type (C3HC4) and AAL25175.1 has a RING-H2 finger.

Table S3 - Phenotypic interaction assay of YabA mutants obtained by saturated mutagenesis at targeted sites (this study).

YabA mutants	Interacting partners		
**AA subtitutions**	**DnaA**	**DnaN**	**YabA**
F62T/Y/H/S/V/C/I/A/L/D/C/G	+	+	+
E75G/Q	+	+	+
Y90F/S/L	-	-	+
Y90C	+	-	+
F94N	+	+/-	+
F94D/P/A/L/G/T	-	+/-	+
F94I/S/V	-	+	+
F94Y	+	+	+
I96N	-	+/-	+
H95L/I	-	-	+
H95Y	-	+	+
H95G	+	+	+
R105V/L/A/T/S/Q/I/H/Y	+	+	+
R105E/G/D	-	+	+
K106V/L/I/T/S/G/E/A/R	+	+	+
E107R/D/T	+	-	+
E107M/L	+	+	+
E107Q/S	+	+/-	+
D108S	+	-	+
D108C	+	+/-	+
F111Y/N/S/A/D	+	-	+
N117L	+	+	+
N117K	+	-	+
K118N/W/A/D/V/P/M/Y/E	+	+	+
K119N/E/Q	+	+	+
K119R	+	-	+

YabA mutant baits were confirmed for the specific loss of one interaction in a yeast two-hybrid assay. The different YabA mutant baits in the yeast haploid strain PJ69-4a (lines) were mated with PJ69-4□ strains containing the preys YabA, DnaA, and DnaN (columns). Interaction phenotypes were monitored using selective media as described in *Materials and Methods*. Mutants lacking the ability to grow onto SC-LUA are indicated by crosses of the corresponding colors

Table S4 - *Bacillus subtilis* strains

Strain	Genotype	Source/construction
JJS142	*JJS39 ΔyabA*	*Noirot-Gros et al., 2006*[a]
JJS138	*JJS39 yabA-N85D (yabA-Aim)*	*Noirot-Gros et al., 2006*[a]
JJS352	*JJS142 amyE::Pxyl-cfp-yabA-F111Y (Spc^R)*	This work
JJS353	*JJS142 amyE::Pxyl-cfp yabA-E107R (Spc^R)*	This work
JJS354	*JJS142 amyE::Pxyl-cfp yabA-E107T (Spc^R)*	This work
JJS355	*JJS142 amyE::Pxyl-cfp yabA-D108S (Spc^R)*	This work
JJS358	*JJS142 amyE::Pxyl-cfp-yabA-F94S (Spc^R)*	This work
JJS359	*JJS142 amyE::Pxyl-cfp-yabA-R105E (Spc^R)*	This work
NIS6052	*yfp-yabA (Cm^R)*	*Hayashi et al., 2005*[b]
MYA002	*ΔyabA::spec yfp-yabA-N85D (Spc^R, Cm^R)*	*Noirot-Gros et al., 2006* [a]
JJS356	*JJS352 yfp-yabA-N85D (Spc^{R}Cm^R)*	This work
JJS357	*JJS354 yfp-yabA-N85D (Spc^{R}Cm^R)*	This work

[a] Noirot-Gros MF, Velten M, Yoshimura M, McGovern S, Morimoto T, Ehrlich SD, Ogasawara N, Polard P, Noirot P. (2006) Functional dissection of YabA, a negative regulator of DNA replication initiation in Bacillus subtilis. *Proc Natl Acad Sci U S A*. Feb 14;103(7):2368-73

[b] Hayashi M, Ogura Y, Harry EJ, Ogasawara N, Moriya S. (2005) Bacillus subtilis YabA is involved in determining the timing and synchrony of replication initiation. *FEMS Microbiol Lett*. Jun 1;247(1):73-9

Table S5: YabA construct primers and coding regions: listed are the primers used for cloning of YabA constructs and their resultant coding regions. Bases written in bold have been appended for annealing or insertion into the YSBLIC3C vector.

Construct	Residues of YabA Encoded	Cloning Primers		Template Used
pET-YSBLIC3C-YabA	1-119	Fwd	5'-**CCAGGGACCAGCAA**TGGATAAAAAAGAGTTATTTGATACAGTCATTAAC-3'	*Bsu*. genomic DNA
		Rev	5'-**GAGGAGAAGGCGCGTTA**TTTTTTATTTAAGAATGACAGACAGAAAAGAC-3'	
pET-YSBLIC3C-YabA$^{1-58}$	1-58	Fwd	5'-**CGACGCAGCAG**TAACGCGCCTTCTCCTCACATATGGC-3'	YSBLIC3C-YabA
		Rev	5'-**AAGGCGCGTTA**CTGCTGCGTCGTATCGTCCAGC-3'	
pET-YSBLIC3C-YabA$^{70-119}$	70-119	Fwd	5'-**AGGGACCAGCAA**AAACACAGAAGACAGAGCAAACTGATATAGGG-3	YSBLIC3C-YabA
		Rev	5'-**GTCTTCTGTGTTTT**TGCTGGTCCCTGGAACAGAACTTCC-3'	
